# Empirical Evidence of Study Design Biases in Randomized Trials: Systematic Review of Meta-Epidemiological Studies

**DOI:** 10.1371/journal.pone.0159267

**Published:** 2016-07-11

**Authors:** Matthew J. Page, Julian P. T. Higgins, Gemma Clayton, Jonathan A. C. Sterne, Asbjørn Hróbjartsson, Jelena Savović

**Affiliations:** 1 School of Social and Community Medicine, University of Bristol, Bristol, United Kingdom; 2 School of Public Health and Preventive Medicine, Monash University, Melbourne, Victoria, Australia; 3 Center for Evidence-Based Medicine, University of Southern Denmark & Odense University Hospital, Odense, Denmark; 4 The National Institute for Health Research Collaboration for Leadership in Applied Health Research and Care West (NIHR CLAHRC West) at University Hospitals Bristol NHS Foundation Trust, Bristol, United Kingdom; Johns Hopkins Bloomberg School of Public Health, UNITED STATES

## Abstract

**Objective:**

To synthesise evidence on the average bias and heterogeneity associated with reported methodological features of randomized trials.

**Design:**

Systematic review of meta-epidemiological studies.

**Methods:**

We retrieved eligible studies included in a recent AHRQ-EPC review on this topic (latest search September 2012), and searched Ovid MEDLINE and Ovid EMBASE for studies indexed from Jan 2012-May 2015. Data were extracted by one author and verified by another. We combined estimates of average bias (e.g. ratio of odds ratios (ROR) or difference in standardised mean differences (dSMD)) in meta-analyses using the random-effects model. Analyses were stratified by type of outcome (“mortality” versus “other objective” versus “subjective”). Direction of effect was standardised so that ROR < 1 and dSMD < 0 denotes a larger intervention effect estimate in trials with an inadequate or unclear (versus adequate) characteristic.

**Results:**

We included 24 studies. The available evidence suggests that intervention effect estimates may be exaggerated in trials with inadequate/unclear (versus adequate) sequence generation (ROR 0.93, 95% CI 0.86 to 0.99; 7 studies) and allocation concealment (ROR 0.90, 95% CI 0.84 to 0.97; 7 studies). For these characteristics, the average bias appeared to be larger in trials of subjective outcomes compared with other objective outcomes. Also, intervention effects for subjective outcomes appear to be exaggerated in trials with lack of/unclear blinding of participants (versus blinding) (dSMD -0.37, 95% CI -0.77 to 0.04; 2 studies), lack of/unclear blinding of outcome assessors (ROR 0.64, 95% CI 0.43 to 0.96; 1 study) and lack of/unclear double blinding (ROR 0.77, 95% CI 0.61 to 0.93; 1 study). The influence of other characteristics (e.g. unblinded trial personnel, attrition) is unclear.

**Conclusions:**

Certain characteristics of randomized trials may exaggerate intervention effect estimates. The average bias appears to be greatest in trials of subjective outcomes. More research on several characteristics, particularly attrition and selective reporting, is needed.

## Introduction

Randomized clinical trials (RCTs) are considered to produce the most credible estimates of the effects of interventions [1–3]. For this reason, they are often used to inform health care and policy decisions, either directly or via their inclusion in systematic reviews. However, intervention effect estimates in RCTs can be biased due to flaws in the design and conduct of the study, which can lead to an overestimation or underestimation of the true intervention effect. Such bias can potentially result in ineffective and harmful interventions being implemented into practice, and effective interventions not being implemented. Authors of systematic reviews of RCTs are therefore encouraged to assess the risk of bias in the included RCTs and to incorporate these assessments into the analysis and conclusions [4].

Empirical evidence can inform which methodological features of RCTs should be considered when appraising RCTs. Many studies have investigated the influence of reported study design characteristics on intervention effect estimates following the landmark study by Schulz et al. [5], which found that trials with inadequate allocation concealment and no double blinding yielded more beneficial estimates of intervention effects. Two syntheses of these studies were recently published. A US Agency for Healthcare Research and Quality (AHRQ) report summarised the results of 38 studies [6]. The authors concluded that some aspects of trial conduct may exaggerate intervention effect estimates, but that most estimates of bias were imprecise and inconsistent between studies. However, they made little distinction between the included studies in terms of their sample size and methodological rigor, and the heterogeneity in average bias estimates within the studies was not examined. A rapid systematic review reached a conclusion similar to the AHRQ review [7], but only three characteristics (sequence generation, allocation concealment and blinding) were examined, while other theoretically important features such as attrition and selective outcome reporting were not.

The aim of this systematic review was to synthesise the results of meta-epidemiological studies that have investigated the average bias and heterogeneity associated with reported methodological features of RCTs.

## Materials and Methods

All methods were pre-specified in a study protocol, which is available in S1 Appendix. This review is reported according to the PRISMA Statement [8] (see S1 PRISMA Checklist).

### Eligibility criteria

#### Types of studies

We included meta-epidemiological studies investigating the association between reported methodological characteristics and intervention effect estimates in RCTs. We considered only meta-epidemiological studies adopting a matched design that ensured that comparisons between trials with different methodological features were only made within the same clinical scenario. Matching is most often done at the meta-analysis level, when a collection of meta-analyses is assembled and the individual trials within each meta-analysis are classified into those with or without a particular methodological characteristic (such as adequate versus inadequate allocation concealment) [9,10]. Matching can also be done at the trial level. For example, a collection of trials is assembled and different measures of the same outcome in each trial are classified into those with or without a characteristic (such as blinded versus unblinded assessment of the same outcome). Or, a multi-arm trial includes a blinded sub-study (such as experimental versus placebo control) and an unblinded sub-study (such as experimental versus no-treatment control) [11]. We included meta-epidemiological studies regardless of the clinical focus (e.g. type of condition, intervention and outcome) or analysis methods used by the investigators.

We excluded single systematic reviews and meta-analyses of RCTs that present a subgroup or sensitivity analysis based on a particular source of bias, since the influence of reported study characteristics on intervention effect estimates tends to be estimated imprecisely within individual meta-analyses. We also excluded studies that assembled a collection of RCTs (e.g. all child health related RCTs published in 2012), and used meta-regression to examine the relationship between a source of bias and trial effect estimates. Such studies do not control for the different interventions examined and outcomes measured across the trials, and so are at high risk of bias due to confounding. Finally, we excluded meta-epidemiological studies comparing randomized with non-randomized studies.

#### Types of methodological features

**Fig 1:**
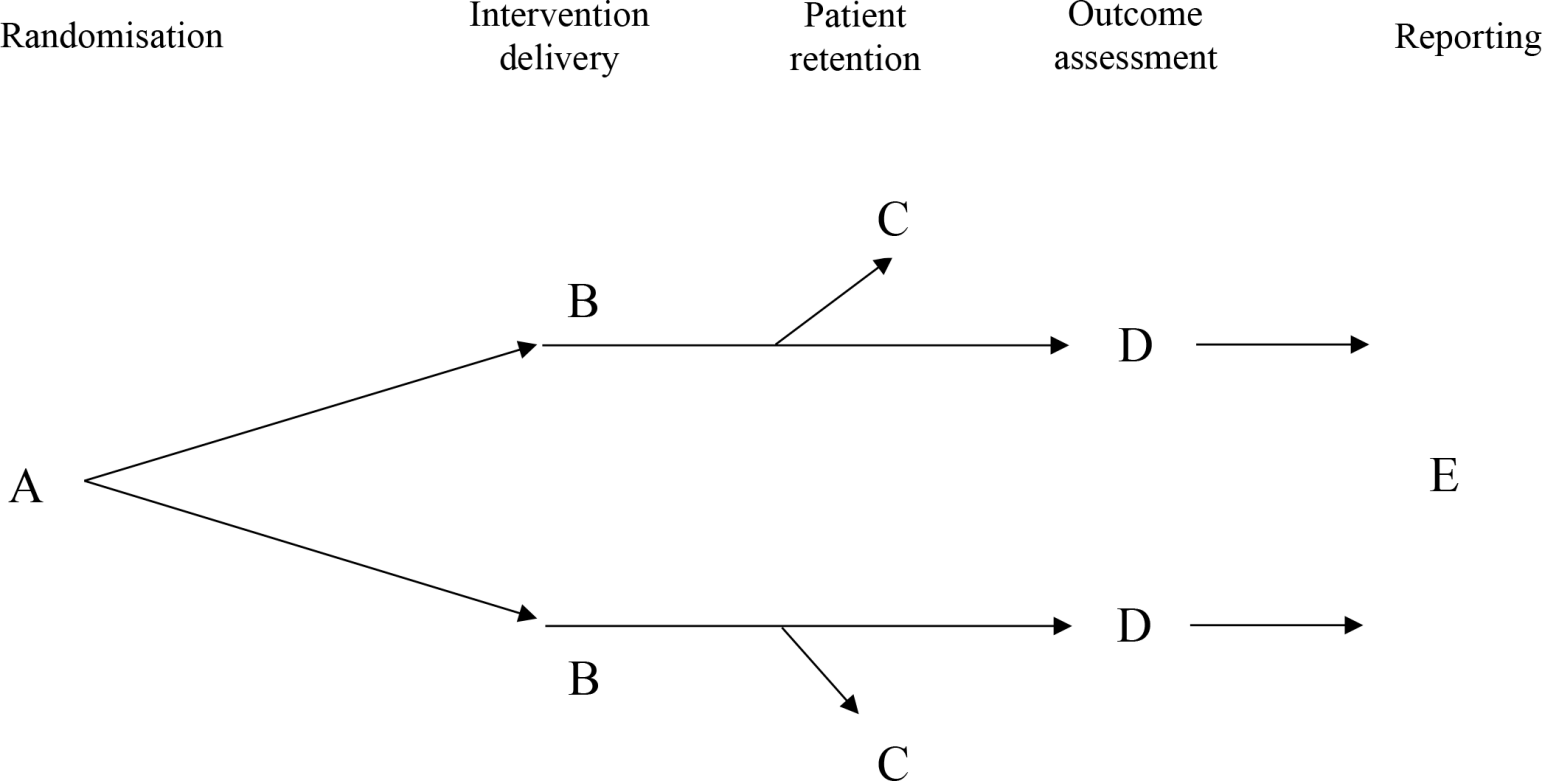
Conceptual framework that underlies the Cochrane risk of bias tool for RCTs. Letters A-E denote the sources of bias listed in Table 1.

We only included meta-epidemiological studies investigating methodological features that can lead to the biases under the conceptual framework that underlies the Cochrane risk of bias tool for RCTs (see Fig 1, Table 1). We included meta-epidemiological studies regardless of how the sources of bias were assessed/defined by the study authors. For example, older meta-epidemiological studies may have used the Jadad scale [12] to assess blinding while more recent meta-epidemiological studies may have used the Cochrane risk of bias tool [13]. Further, some meta-epidemiological studies may have categorised RCTs based on whether “double” or “single” or no blinding was performed, while other studies may have assessed which parties (e.g. patients, trial personnel) were blinded. We excluded meta-epidemiological studies of the association between other characteristics and intervention effect estimates in RCTs (e.g. industry sponsorship [14], sample size [15], single versus multi-centre status [16,17], stopping trials early for benefit or harm [18], and country of enrolment [19]).

**Table 1:** Eligible sources of bias in randomized trials.

Type of bias	Possible methodological features that can lead to bias
A. Bias arising from the randomisation process	Inadequate generation of a random sequence
	Inadequate allocation concealment
	Imbalance in baseline characteristics
	No adjustment for confounding in the analysis
B. Bias due to deviations from intended interventions	Non-blinded participants
	Non-blinded clinician/treatment provider
	Unbalanced delivery of additional interventions or co-interventions
	Participants switching interventions within the trial and being analysed in a group different from the one to which they were randomized
C. Bias due to missing/incomplete outcome	Missing/incomplete outcome data (dropouts, losses to follow-up, or post-randomisation exclusions)
D. Bias in measurement of outcomes	Non-blinded outcome assessor
	Non-blinded data analyst
	Use of faulty measurement instruments (with low validity and reliability)
E. Bias in selection of the reported result	Selective reporting of a subset of outcome domains, or of a subset of outcome measures or analyses for a particular outcome domain.

#### Estimates of interest

Our primary interest was in the association between each methodological characteristic and:

1. the magnitude of the intervention effect estimate (average bias);
2. variation in average bias across meta-analyses (to determine whether average bias estimates are relatively similar or not across meta-analyses addressing different clinical questions), and;
3. the extent of between-trial heterogeneity associated with each characteristic (to determine, for example, whether effect estimates from inadequately concealed trials are more likely to be heterogeneous than estimates from adequately concealed trials). We were also interested in the above estimates stratified by type of outcome (e.g. “mortality” versus “other objective” versus “subjective”) and type of intervention (e.g. “pharmacological” versus “non-pharmacological”), however defined by the study authors. We could not include estimates stratified by type of comparator (e.g. placebo versus no treatment) since such estimates were not reported in any of the included studies. We included meta-epidemiological studies which presented at least one of the estimates of interest.

### Search strategy

We retrieved all meta-epidemiological studies included in the AHRQ report, which searched for studies published up to September 2012 [6]. To identify more recent meta-epidemiological studies, we searched Ovid MEDLINE (Jan 2012 to May 2015) and Ovid EMBASE (Jan 2012 to May 2015). We also searched the Cochrane Database of Systematic Reviews for all reviews edited by the Methodology Review Group (on 20 May 2015), and abstract books of the 2011–2014 Cochrane Colloquia (available at http://abstracts.cochrane.org/) and of the 2011 and 2013 Clinical Trials Methodology Conference (available at http://www.trialsjournal.com/supplements/12/S1/all and http://www.trialsjournal.com/supplements/14/S1/all). Search strategies are presented in S1 Appendix. We reviewed the reference lists of all included meta-epidemiological studies to identify additional meta-epidemiological studies. We also reviewed the list of studies included in two other relevant reviews [7,20].

### Study selection

One reviewer (MJP) screened all titles and abstracts retrieved from the searches. Two reviewers (MJP and GC) independently screened all full text articles retrieved. Any disagreements regarding study eligibility were resolved via discussion

### Data extraction and management

One reviewer (MJP) extracted all of the data using a form developed in Microsoft Excel. A second reviewer (GC) verified the accuracy of all average bias and heterogeneity effect estimates and confidence limits extracted. Data extraction items are presented in S1 Appendix. We did not contact study authors to retrieve any missing data about the study methods and results.

The following data were extracted:

- study characteristics, including the methodological characteristics investigated, how the characteristic was assessed (i.e. number of authors involved in assessment, inter-rater reliability of assessment), definitions of adequate/inadequate characteristics, number of included meta-analyses, number of RCTs included in the meta-analyses, sampling frame (e.g. “random sample of all Cochrane reviews with continuous outcomes that included at least 3 RCTs”), areas of health care addressed, and range of years of publication of the meta-analyses;
- types of outcomes, interventions and comparators examined in the meta-analyses (which were categorised using the classification systems described by Savović et al. [10,21], when sufficient information about each was provided);
- effect estimates and measures of precision (e.g. ratio of odds ratio (ROR) and 95% confidence interval (95% CI);
- any confounding variables assessed by the study authors (e.g. sample size, other methodological characteristics);
- any methods used to deal with potential overlap of RCTs across the meta-analyses.

### Statistical analyses

Characteristics of included meta-epidemiological studies were summarised using frequencies and percentages for binary variables and medians and interquartile ranges (IQRs) for continuous variables.

We analysed the association between a methodological characteristic and the magnitude of an intervention effect estimate (average bias) using the ratio of odds ratios (ROR), ratio of hazard ratios (RHR), or difference in standardised mean differences (dSMD) effect measure, whichever was reported by the study investigators. We analysed the association between a methodological characteristic and between-trial heterogeneity, and the variation in average bias, using the standard deviation of underlying effects (tau) or I^2^. We only analysed associations for each characteristic independently (i.e. we did not consider average bias in trials with *both* inadequate allocation concealment *and* lack of double blinding, or in trials rated at “overall high risk of bias”).

We combined estimates of average bias in a meta-analysis using the random-effects model. We used DerSimonian and Laird’s method of moments estimator to estimate the between-study variance [22]. We assessed statistical inconsistency by inspecting forest plots and calculating the I^2^ statistic [23]. When methodological characteristics were defined differently across the meta-epidemiological studies, we presented average bias effect estimates of each study on forest plots, but did not combine these in a meta-analysis. We presented average bias estimates for all outcomes, subgroups of outcomes (e.g. mortality, other objective, subjective), and subgroups of interventions (e.g. pharmacological, non-pharmacological) where available. To synthesise average bias estimates for binary and continuous outcomes, we converted dSMDs to log RORs by multiplying by π/√3 = 1.814 [24]. The direction of effect was standardised so that a ROR < 1 and dSMD < 0 denotes a larger intervention effect estimate in trials with an inadequate or unclear (versus adequate) characteristic.

Two studies combined data from individual meta-epidemiological studies [10,25]. Wood et al. [25] combined data from three meta-epidemiological studies [5,26,27] while the BRANDO study [10] combined data from these same three meta-epidemiological studies along with four others [28–31]. To avoid double counting we included only the BRANDO estimate in our meta-analyses. The BRANDO investigators ensured that if any meta-analyses appeared in more than one of the seven meta-epidemiological studies, the duplicate meta-analyses were removed (i.e. meta-analyses could not be contributed by more than one of the individual meta-epidemiological studies). We also presented average bias estimates, where available, from the seven contributing meta-epidemiological studies in the forest plots for transparency. Results from Wood et al. are excluded from both forest plots and meta-analyses. Based on the clinical conditions and publication dates of meta-analyses/trials examined in the other meta-epidemiological studies included in our review, we believe that the frequency of overlapping meta-analyses/trials in our meta-analyses is likely to be small.

Some meta-epidemiological studies presented multiple comparisons and analyses for the same outcome. We used the following decision rules to select effect estimates to present in forest plots:

- comparisons selected in the following order: (1) inadequate/unclear versus adequate (or “high/unclear” versus “low” risk of bias); (2) inadequate versus adequate; (3) inadequate versus adequate/unclear.
- adjusted effect estimate selected ahead of unadjusted effect estimate.

## Results

### Results of the search

**Fig 2:**
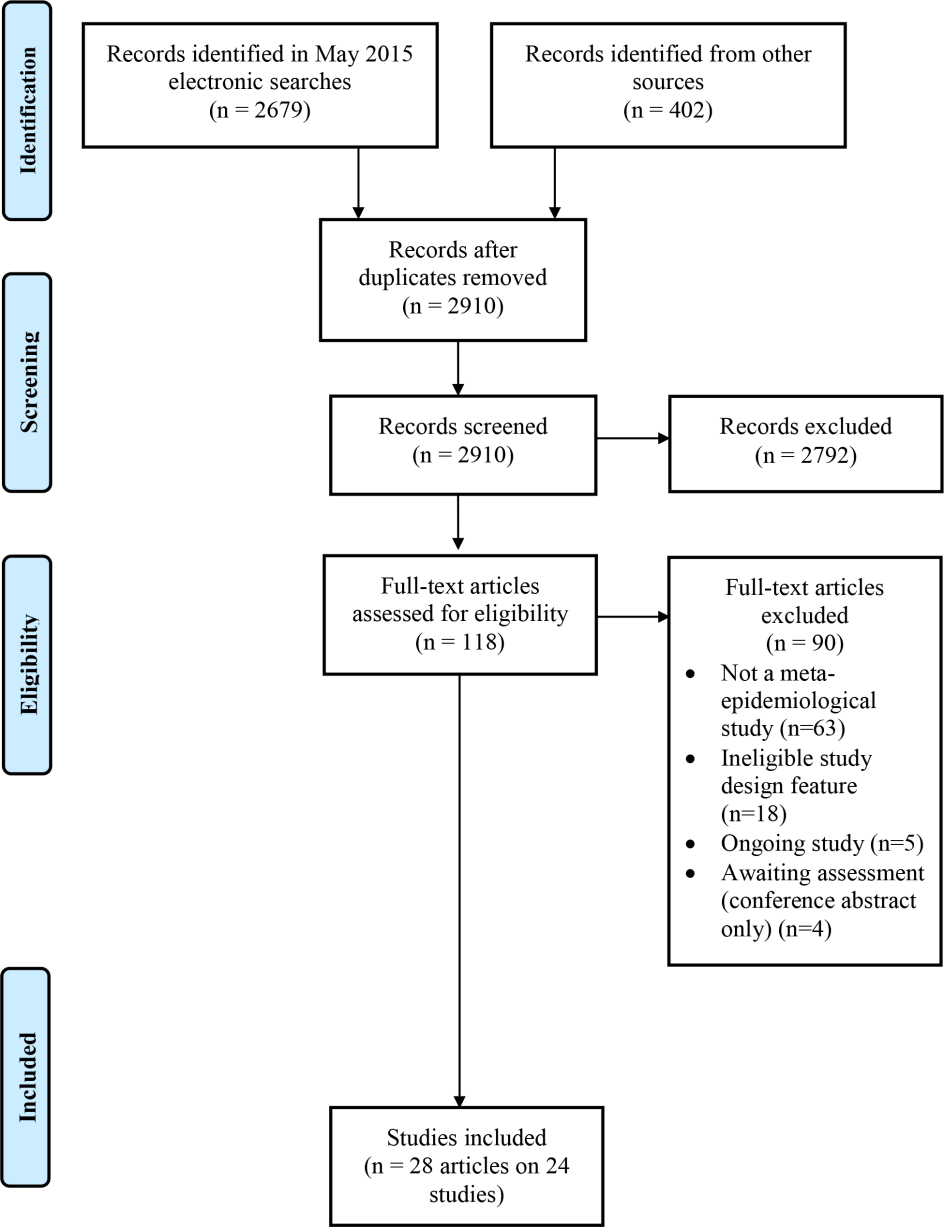
Flow diagram of identification, screening, and inclusion of trials.

A total of 3081 records were identified in the searches. We retrieved 118 full text articles after screening 2910 unique titles/abstracts. Twenty-four meta-epidemiological studies summarised in 28 reports met the inclusion criteria (Fig 2) [5,10,11,21,25–48]. A list of excluded studies is presented in S1 Appendix. Of the 90 excluded studies, the majority were either not meta-epidemiological studies using a matched design or investigated an ineligible study design characteristic. We also identified five ongoing studies [49–53].

### Characteristics of included studies

The included meta-epidemiological studies were published between 1995 and 2015 (Table 2). Matching was done at the meta-analysis level in 20 meta-epidemiological studies (e.g. individual trials within each meta-analysis were classified into those with or without allocation concealment), and at the trial level in four meta-epidemiological studies (e.g. individual outcomes within each trial were classified as measured by a blinded assessor or a non-blinded assessor) [11,39–41]. Meta-epidemiological studies included a median of 26 meta-analyses (published from 1983 to 2014) with a median 229 trials (published from 1955 to 2011). The majority of meta-epidemiological studies included meta-analyses/trials addressing a range of clinical conditions, interventions and outcome types rather than restricting inclusion to a particular clinical area. However, the proportion of each type of condition, intervention and outcome varied considerably across the meta-epidemiological studies (Table 2; characteristics of each individual study are presented in S1 Appendix). The most commonly assessed methodological characteristics were allocation concealment, sequence generation and double blinding. Average bias associated with methodological characteristics was reported in all meta-epidemiological studies. In contrast, increase in between-trial heterogeneity and variation in average bias were reported in only one [21] and 11 meta-epidemiological studies [11,21,32,33,38–41,44–46], respectively. In the majority of meta-epidemiological studies, binary outcomes were analysed, using the meta-meta-analytic approach (where average bias estimates are first derived for each individual meta-analysis, and then combined using a meta-analysis model that can allow for between- and within-meta-analysis heterogeneity) [9]. The issue of non-independence of data (which can occur when the same trial is included in more than one meta-analysis in a study) was avoided or addressed in the analysis in most meta-epidemiological studies (Table 2).

**Table 2:** Summary of characteristics of included meta-epidemiological studies.

Characteristics	Studies (%, n = 24)
***Type of meta-epidemiological study***	
Assembled a collection of meta-analyses, and compared (within each meta-analysis) the effect estimate in trials with versus without a characteristic	20 (83)
Assembled a collection of trials, and compared (within each trial) the effect estimate for the same outcome with versus without a characteristic	3 (13)
Other^**a**^	1 (4)
***Methodological characteristics examined***	
Sequence generation	14 (58)
Allocation concealment	17 (71)
Baseline imbalance	3 (13)
Adjusting for confounders in analysis	1 (4)
Block randomisation in unblinded trials	1 (4)
Blinding of participants	6 (25)
Blinding of personnel	3 (13)
Participants switching intervention groups within the trial	1 (4)
Attrition	10 (42)
Blinding of outcome assessor	7 (29)
Blinding of data analyst	1 (4)
Double blinding	11 (46)
Selective reporting	3 (13)
***Method of assessing methodological characteristics***	
Two reviewers independently assessed all trials	18 (75)
Reliance on assessments by authors of included meta-analyses	4 (17)
One reviewer assessed all trials, with verification by another	1 (4)
Only one author assessed all trials	1 (4)
***Outcomes measured***	
Average bias	24 (100)
Extent of between-trial heterogeneity	1 (5)^**b**^
Variation in average bias	11 (46)
***Number of included meta-analyses/trials***	
Median (IQR) meta-analyses	26 (16–46)
Median (IQR) trials	229 (116–380)
***Year of publication of included meta-analyses/trials***	
Range for meta-analyses	1983–2014
Range for trials	1955–2011
***Area of health care of included meta-analyses/trials***	
Varied	16 (67)
Child/neonatal health only	2 (8)
Osteoarthritis only	2 (8)
Mental health only	1 (4)
Oral medicine only	1 (4)
Pregnancy and childbirth only	1 (4)
Critical care medicine only	1 (4)
***Type of experimental intervention in included meta-analyses/trials***	
Varied (pharmacologic or non-pharmacologic)	21 (88)
Pharmacologic only	1 (4)
Non-pharmacologic only	2 (8)
***Type of outcome in included meta-analyses/trials***	
Varied (mortality, other objective or subjective)	18 (75)
Mortality only	1 (4)
Subjective only	5 (21)
***Type of outcome measure in included meta-analyses/trials***	
Binary	16 (67)
Continuous	7 (29)
Time-to-event	1 (4)
***Analysis approach used*^c^**	
Meta-meta-analytic approach [9]	17 (71)
Logistic regression	4 (17)
Multivariable, multilevel model [47]	3 (13)
Bayesian hierarchical bias model	2 (8)
Bayesian network meta-regression model	1 (4)
No modelling	1 (4)
***How non-independence of data was addressed***	
Dependent trials excluded	12 (50)
Dependent trials included, but analysis adjusted to account for this	6 (25)
Unclear (dependent trials possibly included)	5 (21)
Dependent trials included, with no adjustment for this	1 (4)

All values given as n (%) except where indicated.

^a^ Assembled a collection of trials, and compared (within each trial) the effect estimate in sub-studies with versus without a characteristic. Specifically, investigators included parallel group four-armed clinical trials that randomized patients to a blinded sub-study (experimental vs control) and an otherwise identical nonblind sub-study (experimental vs control). Investigators also included three-armed trials with experimental and no-treatment groups and a placebo group portrayed to patients as another experimental group, so that patients were not informed about the possibility of a placebo intervention. This permitted the experimental group to be included both in a nonblind sub-study (experimental vs no treatment control) and a blind sub-study (experimental vs placebo control)

^b^ Denominator is 20 as between-trial heterogeneity is not applicable in four meta-epidemiological studies

^c^ Percentages do not sum to 100 as some meta-epidemiological studies used more than one approach

### Average bias and heterogeneity associated with methodological characteristics

Estimates of average bias were available for 13 methodological characteristics, of which nine were assessed in more than one meta-epidemiological study (see forest plots in figures below; single study estimates for other characteristics are summarised in the text). Heterogeneity estimates were reported for only six characteristics (Table 3). The criteria used to classify characteristics (i.e. as adequate/unclear/inadequate) were similar across the meta-epidemiological studies for all characteristics except for attrition (definitions used in each study are presented in S1 Appendix). Intervention subgroup estimates (e.g. drug trials versus non-drug trials) of average bias and heterogeneity are presented in S1 Appendix.

**Table 3:** Heterogeneity associated with methodological characteristics.

Study design characteristic	Average bias (95% CI)	Increase in between-trial heterogeneity* (95% CI)	Variation in average bias (95% CI)
**Inadequate/unclear sequence generation (versus adequate)**			
Armijo-Olivo 2015: All outcomes	dSMD -0.02 (-0.15, 0.12)	NR	tau 0.10
BRANDO (Savović 2012): All outcomes	ROR 0.90 (0.82, 0.99)	tau 0.06 (0.01, 0.20)	tau 0.05 (0.01, 0.15)
BRANDO (Savović 2012): Mortality	ROR 0.86 (0.69, 1.06)	tau 0.08 (0.01, 0.31)	tau 0.06 (0.01, 0.28)
BRANDO (Savović 2012): Other objective	ROR 1.00 (0.84, 1.20)	tau 0.07 (0.01, 0.30)	tau 0.07 (0.01, 0.27)
BRANDO (Savović 2012): Subjective	ROR 0.88 (0.76, 1.00)	tau 0.05 (0.01, 0.21)	tau 0.06 (0.01, 0.24)
Papageorgiou 2015: All outcomes	dSMD -0.01 (-0.26, 0.25)	NR	tau 0.46
**Inadequate/unclear allocation concealment (versus adequate)**			
Armijo-Olivo 2015: All outcomes	dSMD -0.12 (-0.30, 0.06)	NR	tau 0.21
BRANDO (Savović 2012): All outcomes	ROR 0.89 (0.81, 0.99)	tau 0.06 (0.01, 0.19)	tau 0.05 (0.01, 0.18)
BRANDO (Savović 2012): Mortality	ROR 1.03 (0.82, 1.31)	tau 0.07 (0.01, 0.30)	tau 0.07 (0.01, 0.33)
BRANDO (Savović 2012): Other objective	ROR 0.92 (0.76, 1.12)	tau 0.06 (0.01, 0.24)	tau 0.06 (0.01, 0.29)
BRANDO (Savović 2012): Subjective	ROR 0.82 (0.70, 0.94)	tau 0.08 (0.01, 0.27)	tau 0.07 (0.01, 0.30)
Herbison 2011: All outcomes	ROR 0.91 (0.83, 0.99)	NR	tau 0.19
Nuesch 2009a: Subjective outcomes	dSMD -0.15 (-0.31, 0.02)	NR	tau 0.24
**Lack of/unclear blinding of participants (versus blinding)**			
Hrobjartsson 2014b: Subjective	dSMD -0.56 (-0.71, -0.41)	NA	I^2^ 60%
Nuesch 2009a: Subjective	dSMD -0.15 (-0.39, 0.09)	NR	tau 0.26
**Lack of/unclear blinding of outcome assessor (versus blinding)**			
Hrobjartsson 2012: Subjective	ROR 0.64 (0.43, 0.96)	NA	I^2^ 45%
Hrobjartsson 2013: Subjective	dSMD -0.23 (-0.40, -0.06)	NA	I^2^ 46%
Hrobjartsson 2014a: Subjective (standard trials)	RHR 0.73 (0.57, 0.93)	NA	I^2^ 24%
Hrobjartsson 2014a: Subjective (atypical trials)	RHR 1.33 (0.98, 1.82)	NA	I^2^ 0%
**Lack of/unclear double blinding (versus double blinding)**			
BRANDO (Savović 2012): All outcomes	ROR 0.86 (0.73, 0.98)	tau 0.20 (0.02, 0.39)	tau 0.17 (0.03, 0.32)
BRANDO (Savović 2012): Mortality	ROR 1.07 (0.78, 1.48)	tau 0.09 (0.01, 0.44)	tau 0.08 (0.01, 0.42)
BRANDO (Savović 2012): Other objective	ROR 0.91 (0.64, 1.33)	tau 0.10 (0.01, 0.50)	tau 0.20 (0.02, 0.85)
BRANDO (Savović 2012): Subjective	ROR 0.77 (0.61, 0.93)	tau 0.24 (0.02, 0.45)	tau 0.20 (0.04, 0.39)
**Attrition (versus no or minimal attrition)**			
Abraha 2015: All outcomes	ROR 0.80 (0.69, 0.94)	NR	tau 0.28
Abraha 2015: Objective	ROR 0.80 (0.60, 1.06)	NR	tau 0.42
Abraha 2015: Subjective	ROR 0.84 (0.70, 1.01)	NR	tau 0.33
BRANDO (Savović 2012): All outcomes	ROR 1.07 (0.92, 1.25)	tau 0.07 (0.01, 0.24)	tau 0.06 (0.01, 0.24)
BRANDO (Savović 2012): Mortality	ROR 1.07 (0.80, 1.42)	tau 0.10 (0.01, 0.32)	tau 0.09 (0.01, 0.75)
BRANDO (Savović 2012): Other objective	ROR 1.35 (0.63, 2.94)	tau 0.13 (0.01, 1.05)	tau 0.13 (0.01, 1.15)
BRANDO (Savović 2012): Subjective	ROR 1.03 (0.79, 1.36)	tau 0.07 (0.01, 0.38)	tau 0.07 (0.01, 0.35)

* tau is on the log scale for RORs, but not for dSMDs

CI = confidence interval; dSMD = difference in standardised mean differences; NA = not applicable; NR = not reported; RHR = ratio of hazard ratios; ROR = ratio of odds ratios. dSMD < 0 and ROR and RHR < 1 = larger effect in trials with inadequate characteristic (or at high/unclear risk of bias)

#### Bias arising from the randomisation process

**Fig 3:**
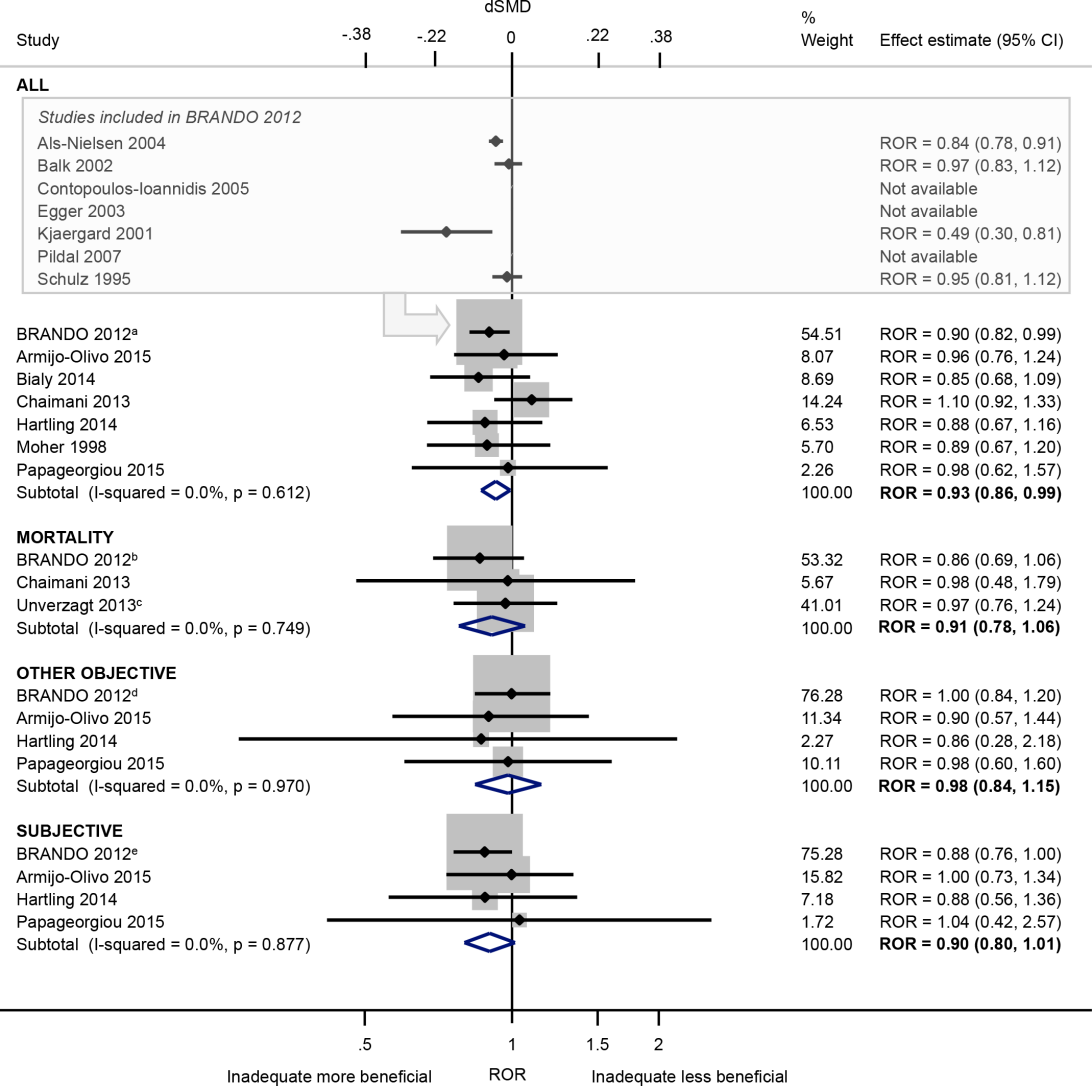
Random-effects meta-analysis of RORs associated with inadequate/unclear (versus adequate) sequence generation. The boxed section displays the average bias estimates, where available, from the seven meta-epidemiological studies contributing to the BRANDO 2012^a^ study (however only the BRANDO 2012^a^ ROR was included in our meta-analysis). The BRANDO 2012^a^ ROR is based on a multivariable analysis with adjustment for allocation concealment and double blinding [the corresponding univariable ROR is (95% CrI) 0.89 (0.82, 0.96)]. The BRANDO 2012^b^ ROR is based on a multivariable analysis with adjustment for allocation concealment and double blinding [the corresponding univariable ROR (95% CrI) is 0.89 (0.75, 1.05)]. The Unverzagt 2013^c^ ROR is based on a multivariable analysis with adjustment for allocation concealment, double blinding, attrition, selective outcome reporting, early stopping, pre-intervention, competing interests, baseline imbalance, switching interventions, sufficient follow-up, and single- versus multi-centre status [the corresponding univariable ROR (95% CI) is 0.98 (0.8, 1.21)]. The BRANDO 2012^d^ ROR is based on a multivariable analysis with adjustment for allocation concealment and double blinding [the corresponding univariable ROR (95% CrI) is 0.99 (0.84, 1.16)]. The BRANDO 2012^e^ ROR is based on a multivariable analysis with adjustment for allocation concealment and double blinding [the corresponding univariable ROR (95% CrI) is 0.83 (0.74, 0.94)].

Based on a meta-analysis of seven meta-epidemiological studies [21,33–35,37,42,46], inadequate/unclear (versus adequate) sequence generation was associated with a 7% exaggeration of intervention effect estimates on average (ROR 0.93, 95% CI 0.86 to 0.99; I^2^ 0%; Fig 3). The bias appears to be greater in trials of subjective outcomes (ROR 0.90, 95% CI 0.80 to 1.01; I^2^ 0%; 4 meta-epidemiological studies [21,33,37,46]) compared with trials of other objective outcomes (ROR 0.98, 95% CI 0.84 to 1.15; I^2^ 0%; 4 meta-epidemiological studies [21,33,37,46]), although the 95% CIs overlap. Inadequate/unclear (versus adequate) sequence generation led to only a small increase in between-trial heterogeneity within the meta-analyses in the BRANDO study. The variation in average bias across meta-analyses was minimal in two meta-epidemiological studies [21,33], but high in the study of oral medicine meta-analyses [46] (Table 3).

**Fig 4:**
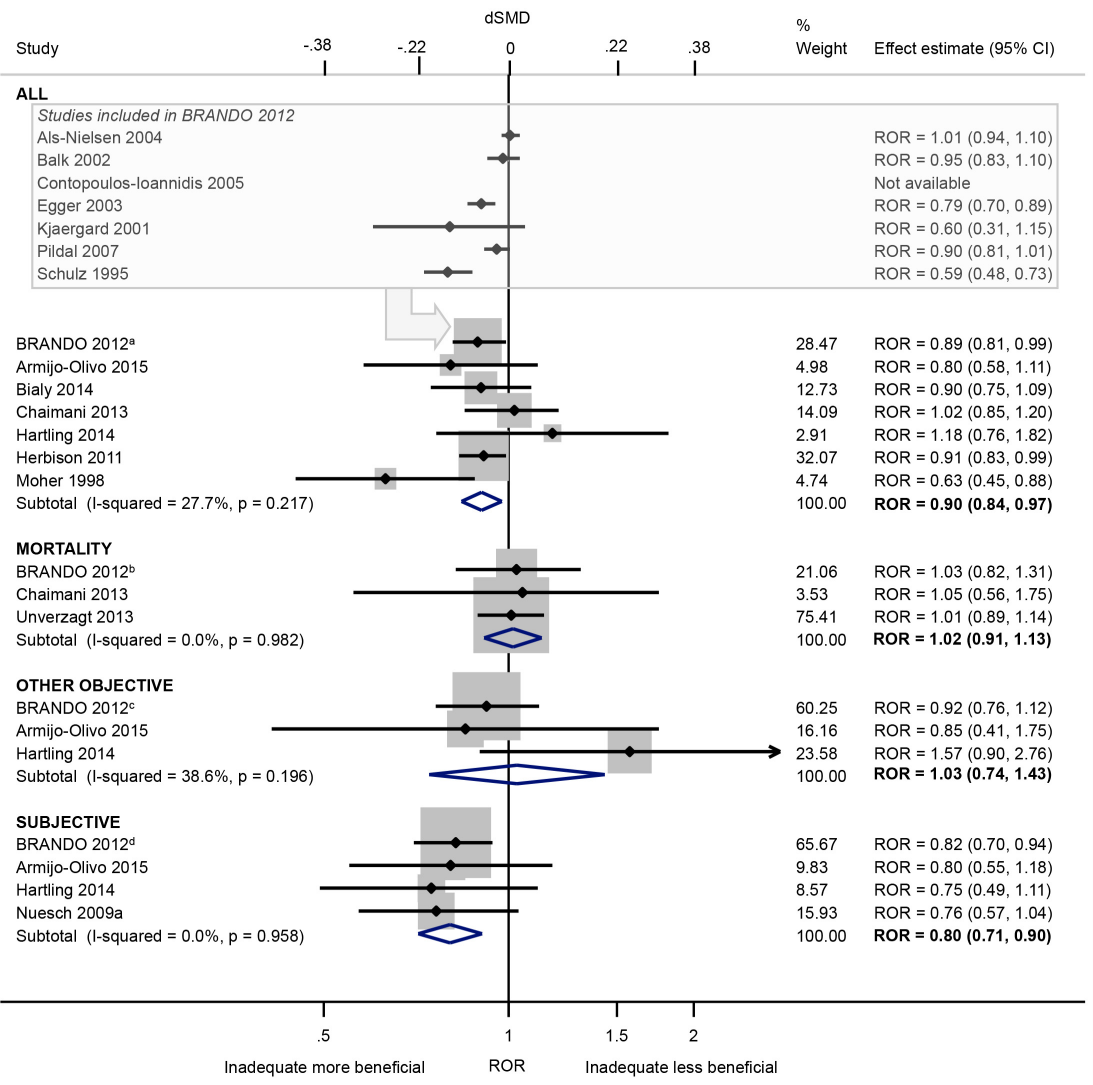
Random-effects meta-analysis of RORs associated with inadequate/unclear (versus adequate) allocation concealment. The boxed section displays the average bias estimates, where available, from the seven meta-epidemiological studies contributing to the BRANDO 2012^a^ study (however only the BRANDO 2012^a^ ROR was included in our meta-analysis). The BRANDO 2012^a^ ROR is based on a multivariable analysis with adjustment for sequence generation and double blinding [the corresponding univariable ROR (95% CrI) is 0.93 (0.87, 0.99)]. The BRANDO 2012^b^ ROR is based on a multivariable analysis with adjustment for sequence generation and double blinding [the corresponding univariable ROR (95% CrI) is 0.98 (0.88, 1.10)]. The BRANDO 2012^c^ ROR is based on a multivariable analysis with adjustment for sequence generation and double blinding [the corresponding univariable ROR (95% CrI) is 0.97 (0.85, 1.10)]. The BRANDO 2012^d^ ROR is based on a multivariable analysis with adjustment for sequence generation and double blinding [the corresponding univariable ROR (95% CrI) is 0.85 (0.75, 0.95)].

Our meta-analysis of seven meta-epidemiological studies [21,33–35,37,38,42] suggests that intervention effect estimates tends to be exaggerated by 10% in trials with inadequate/unclear (versus adequate) allocation concealment (ROR 0.90, 95% CI 0.84 to 0.97; I^2^ 28%; Fig 4). The average bias was greatest in trials of subjective outcomes (ROR 0.80, 95% CI 0.71 to 0.90; I^2^ 0%; 4 meta-epidemiological studies [21,33,37,44]), and in trials of complementary and alternative medicine interventions (CAM) (Dsmd -0.52 versus -0.01 in non-CAM trials; 1 meta-epidemiological study [44]; S1 Appendix). Little evidence of bias in trials of mortality or other objective outcomes was observed (ROR 1.02 and 1.03, respectively). There was only a limited increase in between-trial heterogeneity and limited variation in average bias in the BRANDO study, whereas variation in average bias was high in three smaller meta-epidemiological studies [33,38,44] (Table 3).

**Fig 5:**
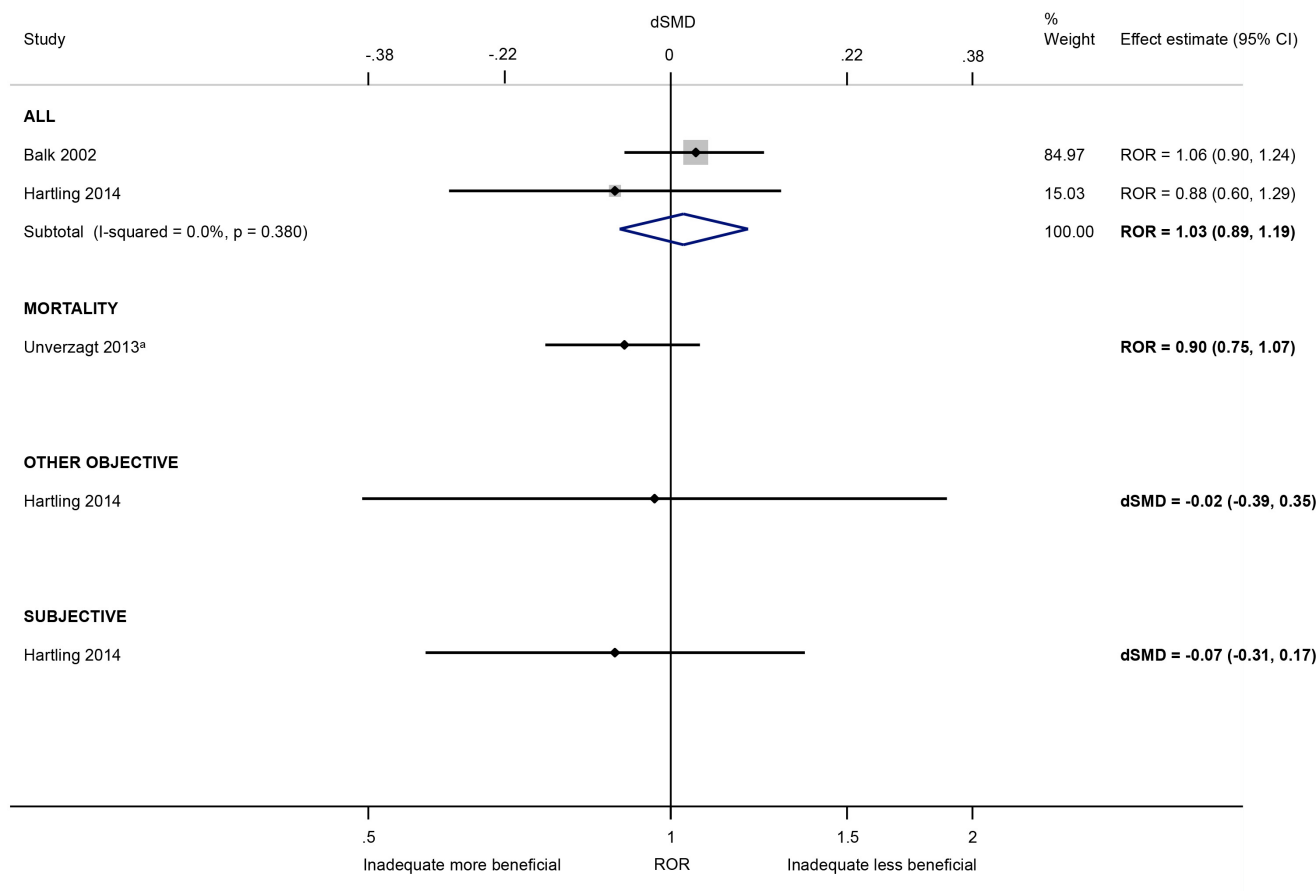
Random-effects meta-analysis of RORs and dSMDs associated with presence (versus absence) of baseline imbalance. The Unverzagt 2013^a^ ROR is based on a multivariable analysis with adjustment for sequence generation, allocation concealment, double blinding, attrition, selective outcome reporting, early stopping, pre-intervention, competing interests, switching interventions, sufficient follow-up, and single- versus multi-centre status [the corresponding univariable ROR (95% CI) is 0.92 (0.80, 1.06)].

The influence of other sources of bias arising from the randomisation process were less clear. There was little evidence that the presence (versus absence) of baseline imbalance inflates intervention effects (ROR 1.03, 95% CI 0.89 to 1.19; I^2^ 0%; 2 meta-epidemiological studies [29,37]; Fig 5); this lack of association was found regardless of the type of outcome, but all estimates were very imprecise. Also, there was little evidence that intervention effect estimates were exaggerated in trials without (versus with) adjustment for confounders (ROR 0.96, 95% CI 0.79 to 1.23; 1 meta-epidemiological study [29]), or which used (versus did not use) block randomisation in unblinded trials (dSMD -0.18, 95% CI -0.47 to 0.11; 1 meta-epidemiological study [37]). However, each characteristic was only examined in a single small meta-epidemiological study (with at most 26 meta-analyses).

#### Bias due to deviations from intended interventions

**Fig 6:**
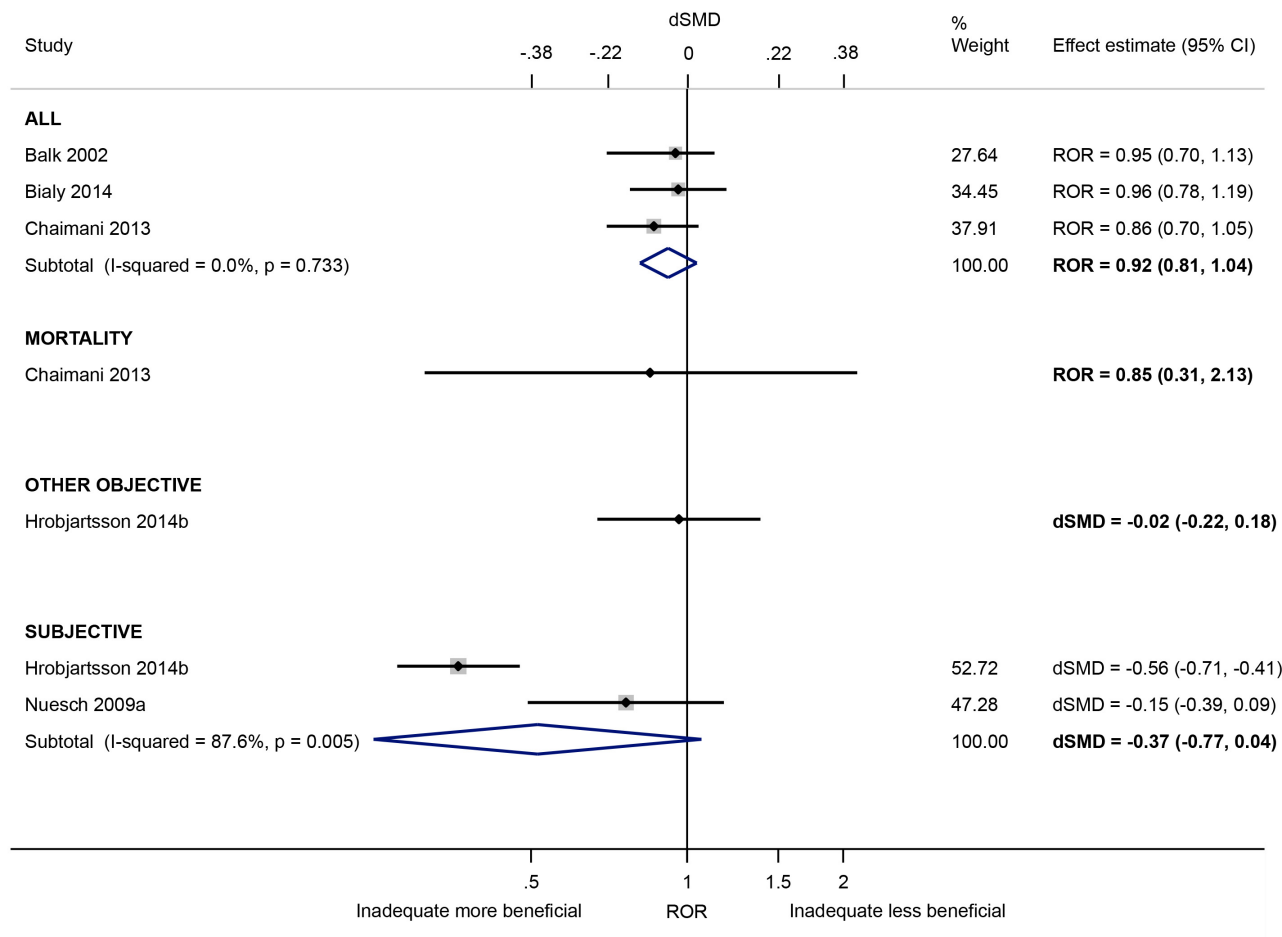
Random-effects meta-analysis of RORs and dSMDs associated with lack of/unclear blinding of participants (versus blinding of participants).

Based on a meta-analysis of three meta-epidemiological studies [29,34,35] examining objective and subjective outcomes together, there was little evidence of bias in trials with lack of/unclear blinding of participants (versus blinding of participants) (ROR 0.92, 95% CI 0.81 to 1.04; I^2^ 0%; Fig 6)). No association was also found in single meta-epidemiological studies examining trials of mortality [35] or other objective outcomes [41]. However, intervention effects appear to be exaggerated in trials with subjectively measured outcomes (dSMD -0.37, 95% CI -0.77 to 0.04; I^2^ 88%; 2 meta-epidemiological studies [41,44]). The average bias was larger in the meta-epidemiological study by Hrobjartsson et al. (dSMD -0.56) compared with Nuesch et al. (dSMD -0.15), in acupuncture trials (dSMD -0.63) versus non-acupuncture trials (dSMD -0.17), and in non-drug trials (dSMD -0.67) versus drug trials (dSMD 0.04) (S1 Appendix). Inconsistency in average bias was moderate in one meta-epidemiological study (I^2^ 60% [41]) and the magnitude of heterogeneity was high in another (tau 0.26 [44]) (Table 3).

**Fig 7:**
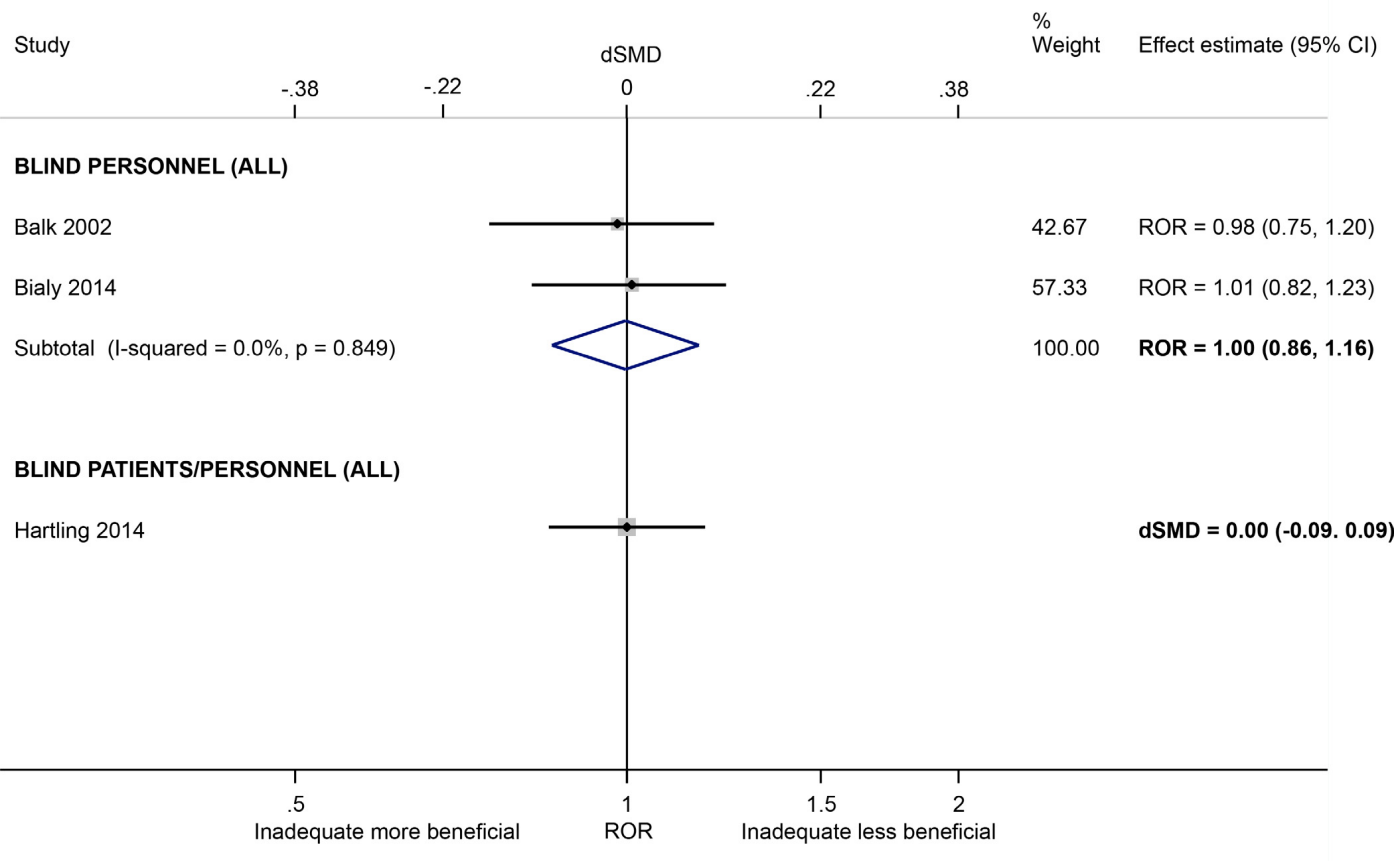
Random-effects meta-analysis of RORs and dSMDs associated with lack of/unclear blinding of personnel or participants/personnel (versus blinding of either party).

Intervention effect estimates for binary outcomes were not exaggerated in trials with lack of/unclear blinding of personnel (versus blinding of personnel) (ROR 1.00, 95% CI 0.86 to 1.16; I^2^ 0%; 2 meta-epidemiological studies [29,34]; Fig 7). A similar lack of effect on continuous outcomes was found in trials with lack of/unclear blinding of participants *or* personnel (versus blinding of either party) (dSMD 0.00, 95% CI -0.09 to 0.09; 1 meta-epidemiological study [37]). However, all three meta-epidemiological studies were small and two focused on meta-analyses in only one clinical area, so the results may have limited generalisability.

Bias due to participants switching interventions within the trial and being analysed in a group different from the one to which they were randomized was examined in one small meta-epidemiological study of 12 meta-analyses in critical care medicine [48]. The ROR for mortality effect estimates was 0.89 (95% CI 0.61 to 1.31).

#### Bias due to missing/incomplete outcome data

**Fig 8:**
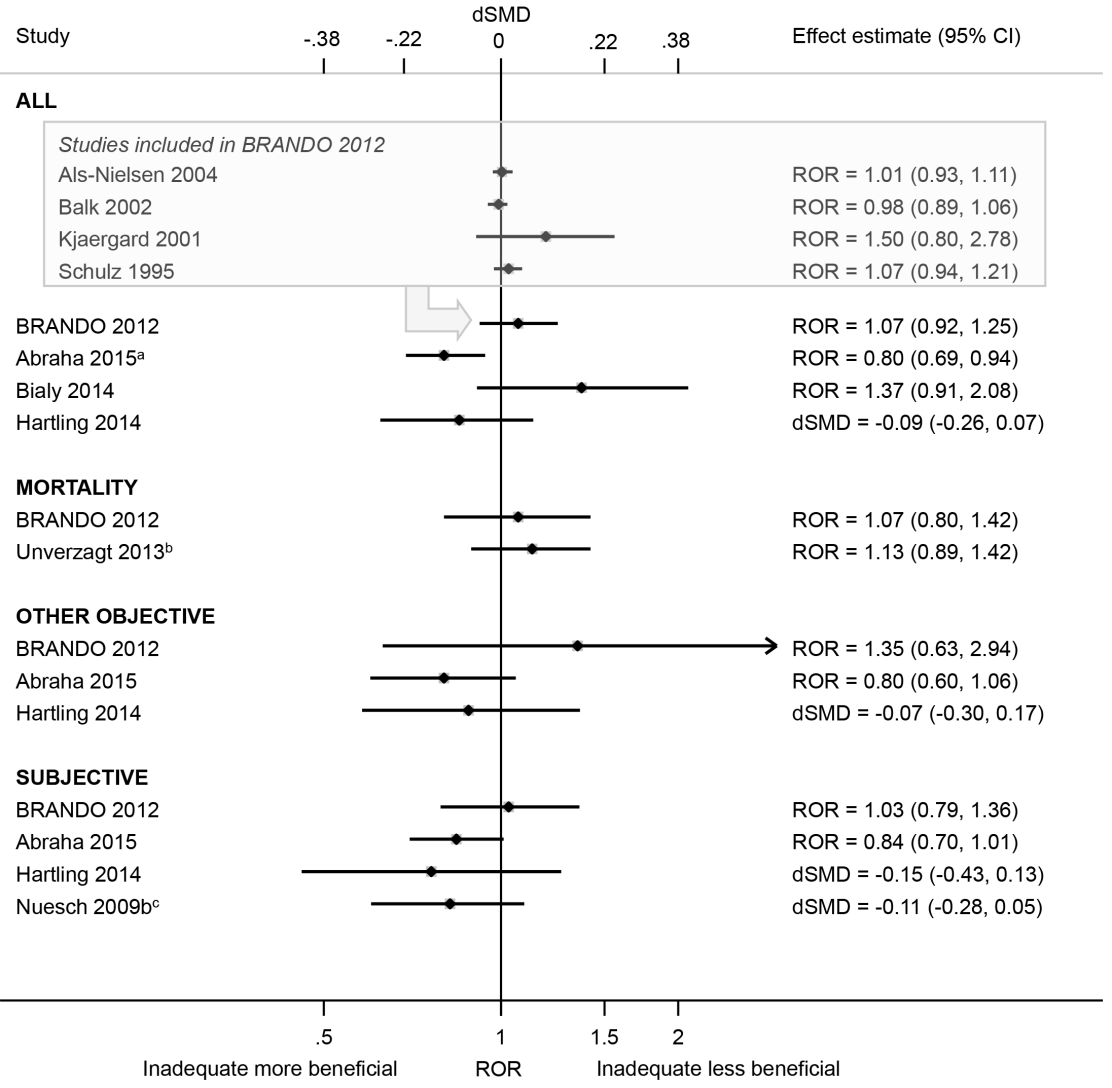
Estimated RORs and dSMDs associated with any (versus no or minimal) attrition. The boxed section displays the average bias estimates, where available, from the four meta-epidemiological studies contributing to the BRANDO 2012 study. The Abraha 2015^a^ ROR is based on a multivariable analysis with adjustment for use of placebo comparison, sample size, type of centre, items of risk of bias, post-randomisation exclusions, funding, and publication bias [the corresponding univariable ROR (95% CI) is 0.83 (0.71, 0.97)]. The Unverzagt 2013^b^ ROR is based on a multivariable analysis with adjustment for sequence generation, allocation concealment, double blinding, selective outcome reporting, early stopping, pre-intervention, competing interests, baseline imbalance, switching interventions, sufficient follow-up, and single- versus multi-centre status [the corresponding univariable ROR (95% CI) is 1.19 (0.98, 1.45)]. The Nuesch 2009b^c^ dSMD is based on a multivariable analysis with adjustment for allocation concealment [the corresponding multivariable dSMD (95% CI) with adjustment for blinding of participants is -0.15 (-0.30, 0.00), and the corresponding univariable dSMD (95% CI) is -0.13 (-0.29, 0.04)].

We did not combine estimates of average bias due to attrition because the definition of attrition varied across the meta-epidemiological studies (see S1 Appendix). Attrition was associated with overestimation of effect estimates in some meta-epidemiological studies and underestimation in others, regardless of the type of outcome (Fig 8). For example, reporting the use of a “modified” intention-to-treat (mITT) analysis (versus ITT) was associated with exaggeration of intervention effect estimates (ROR 0.80, 95% CI 0.69 to 0.94; 1 meta-epidemiological study [32]), but having a dropout rate >20% (versus ≤20%) was not (ROR 1.07, 95% CI 0.92 to 1.25; 1 meta-epidemiological study [21]). The variation in average bias estimates across meta-analyses also differed between the meta-epidemiological studies (Table 3).

#### Bias in measurement of outcomes

**Fig 9:**
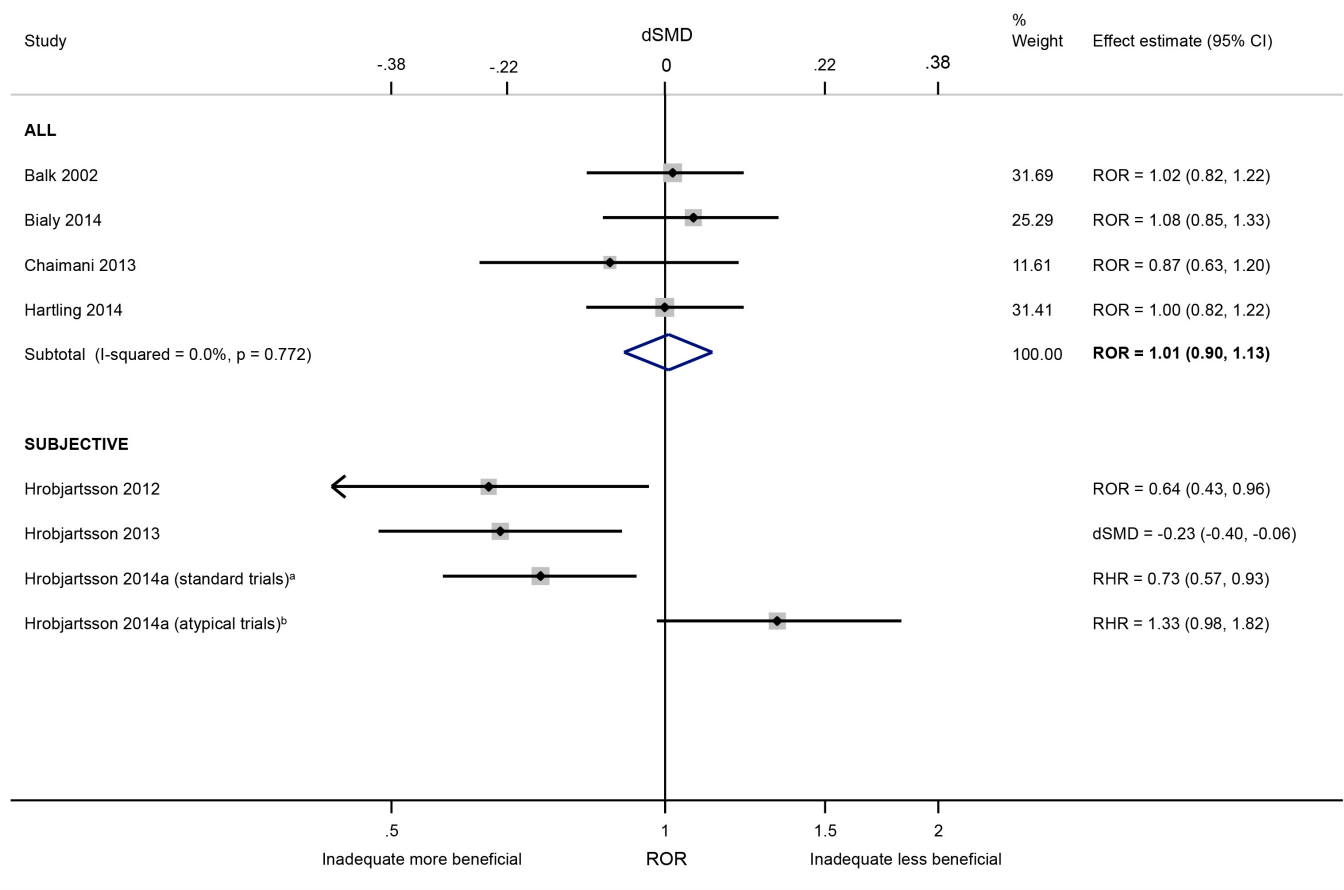
Random-effects meta-analysis of RORs and dSMDs associated with lack of/unclear blinding of outcome assessors (versus blinding of outcome assessors). RHR = Ratio of hazard ratios. Hróbjartsson 2014a^a^ “standard trials” comprise those comparing experimental interventions with standard control interventions, such as placebo, no-treatment, usual care or active control. Hróbjartsson 2014a^b^ “atypical trials” comprise those comparing an oral experimental administration of a drug with the intravenous control administration of the same drug for cytomegalovirus retinitis.

The influence of lack of/unclear blinding of outcome assessors (versus blinding) was negligible in a meta-analysis of four meta-epidemiological studies [29,34,35,37] which analysed objective and subjective outcomes together (ROR 1.01, 95% CI 0.90 to 1.13; I^2^ 0%; Fig 9). In contrast, intervention effect estimates were exaggerated in trials with unblinded (versus blinded) assessment of subjective binary (ROR 0.64, 95% CI 0.43 to 0.96; 1 meta-epidemiological study [11]), continuous (dSMD -0.23, 95% CI -0.40 to -0.06; 1 meta-epidemiological study [39]) and time-to-event outcomes (RHR 0.73, 95% CI 0.57 to 0.93; 1 meta-epidemiological study [40]). There was moderate inconsistency in average bias in these three meta-epidemiological studies (I^2^ range 24% to 46%) (Table 3).

**Fig 10:**
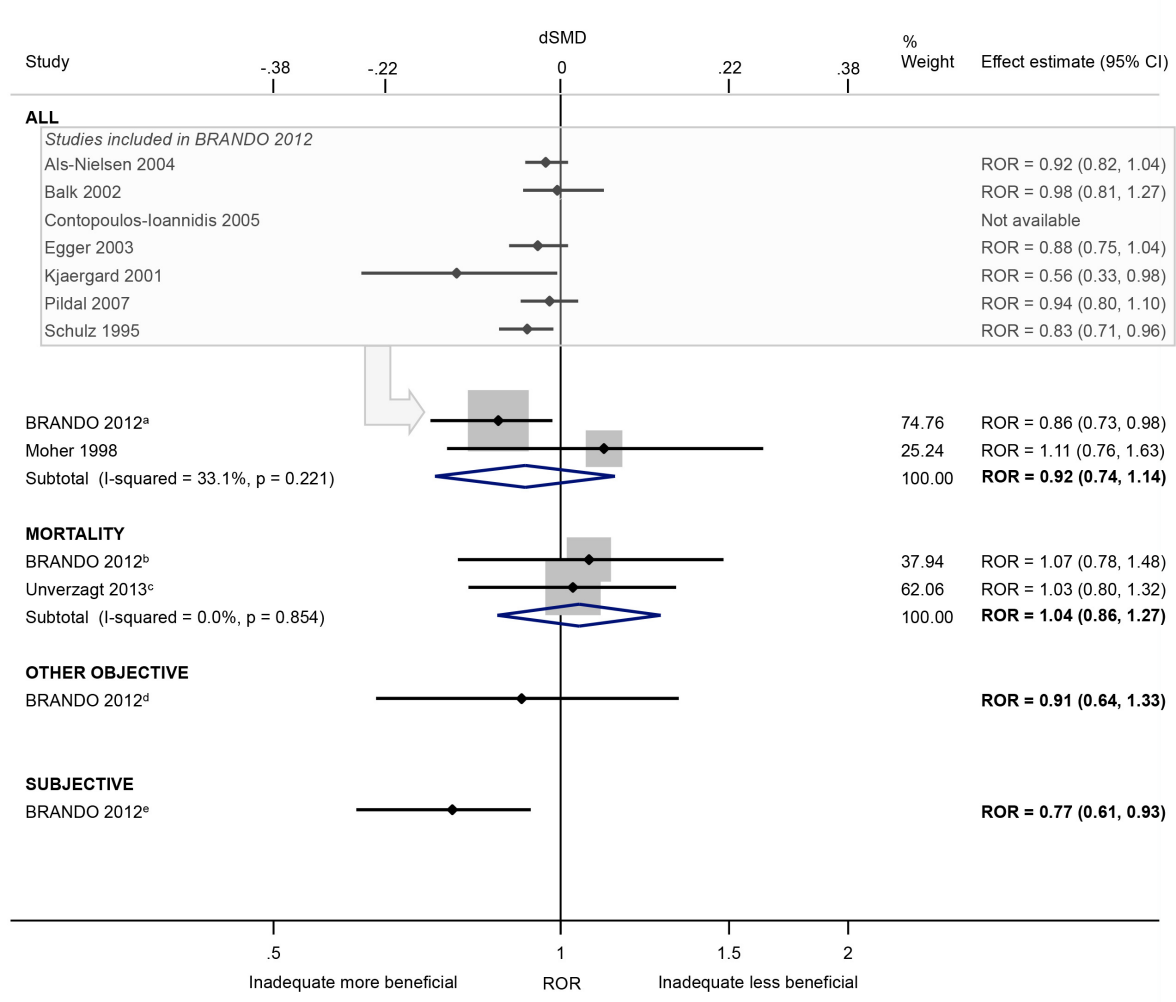
Random-effects meta-analysis of RORs associated with lack of/unclear double blinding (versus double blinding). The boxed section displays the average bias estimates, where available, from the seven meta-epidemiological studies contributing to the BRANDO 2012^a^ study (however only the BRANDO 2012^a^ ROR was included in our meta-analysis). The BRANDO 2012^a^ ROR is based on a multivariable analysis with adjustment for sequence generation and allocation concealment [the corresponding univariable ROR (95% CrI) is 0.87 (0.79, 0.96)]. The BRANDO 2012^b^ ROR is based on a multivariable analysis with adjustment for sequence generation and allocation concealment [the corresponding univariable ROR (95% CrI) is 0.92 (0.80, 1.04)]. The Unverzagt 2013^c^ ROR is based on a multivariable analysis with adjustment for sequence generation, allocation concealment, attrition, selective outcome reporting, early stopping, pre-intervention, competing interests, baseline imbalance, switching interventions, sufficient follow-up, and single- versus multi-centre status [the corresponding univariable ROR (95% CI) is 0.84 (0.69, 1.02)]. The BRANDO 2012^d^ ROR is based on a multivariable analysis with adjustment for sequence generation and allocation concealment [the corresponding univariable ROR (95% CrI) is 0.93 (0.74, 1.18)]. The BRANDO 2012^e^ ROR is based on a multivariable analysis with adjustment for sequence generation and allocation concealment [the corresponding univariable ROR (95% CrI) is 0.78 (0.65, 0.92)].

Lack of/unclear double blinding (versus double blinding, where both participants and personnel/assessors are blinded) was associated with a 23% exaggeration of intervention effect estimates in trials with subjective outcomes (ROR 0.77, 95% CI 0.61 to 0.93; 1 meta-epidemiological study [21]). In contrast, there was little evidence of such bias in trials of mortality or other objective outcomes, or when all outcomes were analysed (ROR 0.92, 95% CI 0.74 to 1.14; I^2^ 33%; 2 meta-epidemiological studies [21,42]; Fig 10). In the BRANDO study, there was an increase in between-trial heterogeneity in trials with no/unclear (versus clear) double blinding, and the average bias varied between meta-analyses (Table 3).

In one meta-epidemiological study, blinding of data analysts was recorded, but average bias could not be quantified because the number of informative meta-analyses (i.e. those including trials with and without the characteristic) was too low [29]. No meta-epidemiological study examined bias due to use of faulty measurement instruments (with low validity and reliability).

#### Bias in selection of the reported result

**Fig 11:**
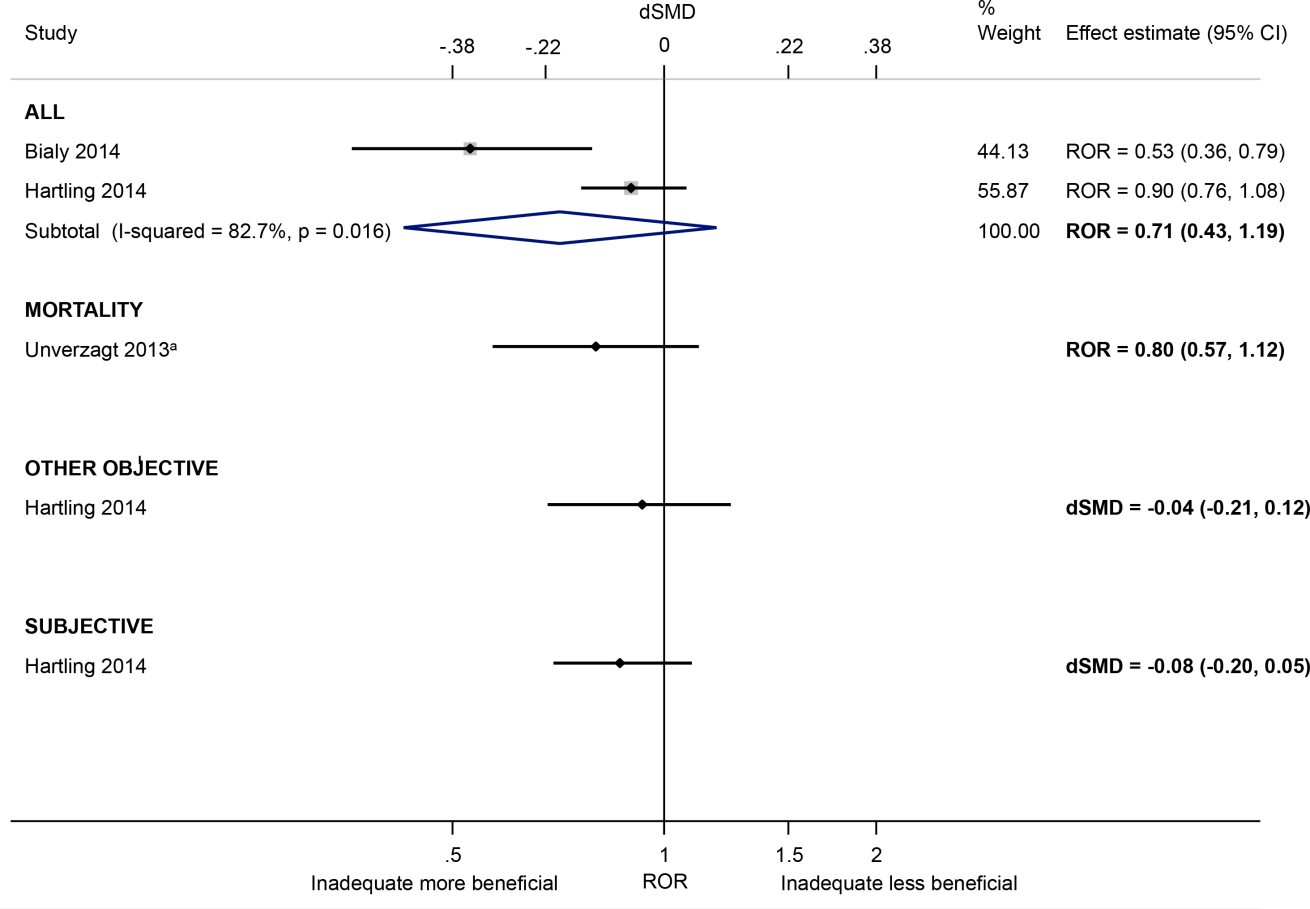
Random-effects meta-analysis of RORs and dSMDs associated with high/unclear (versus low) risk of bias due to selective reporting. The Unverzagt 2013^a^ ROR is based on a multivariable analysis with adjustment for sequence generation, allocation concealment, double blinding, attrition, early stopping, pre-intervention, competing interests, baseline imbalance, switching interventions, sufficient follow-up, and single- versus multi-centre status [the corresponding univariable ROR (95% CI) is 0.73 (0.54, 0.98)].

Based on a meta-analysis of two small meta-epidemiological studies [34,37], there was no convincing evidence that trials rated at high/unclear (versus low) risk of bias due to selective reporting have larger effect estimates (ROR 0.71, 95% CI 0.43 to 1.19; Fig 11), but the inconsistency in estimates was high (I^2^ 83%). Trials were only rated at high risk of bias if any outcome domain was inconsistent between the methods and results section. This differs from the scenario where the reported effect estimate has been selected from among multiple measures or analyses (e.g. trialists perform multiple adjusted analyses yet only report that which yielded the most favourable effect). Such bias in selection of the reported result was not investigated in any of the included meta-epidemiological studies.

## Discussion

This review of 24 meta-epidemiological studies suggests that on average, intervention effect estimates are exaggerated in trials with inadequate/unclear (versus adequate) sequence generation and allocation concealment. For these characteristics, the average bias appears to be larger in trials of subjective outcomes compared with other objective outcomes. For subjective outcomes, intervention effect estimates appear to be exaggerated in trials with lack of/unclear blinding of participants (versus blinding of participants), lack of/unclear blinding of outcome assessors (versus blinding of outcome assessors) and lack of/unclear double blinding (versus double blinding, where both participants and personnel/assessors are blinded). The average bias due to attrition varied depending on how it was defined. The influence of other characteristics (baseline imbalance, no adjustment for confounders, use of block randomisation in unblinded trials, unblinded personnel, and analysing participants in a group different from the one to which they were randomized) is uncertain, because they have been examined in only a few small meta-epidemiological studies. Some characteristics have not been investigated in any meta-epidemiological study (unblinded data analysts, use of faulty measurement instruments, bias in selection of the reported results). Only one meta-epidemiological study measured the between-trial heterogeneity associated with characteristics [21], which was increased in trials without double blinding, but less so in trials with inadequate/unclear sequence generation, allocation concealment and attrition. The average bias estimates within meta-epidemiological studies examining the impact of sequence generation, allocation concealment, patient blinding, outcome assessor blinding, double blinding and attrition varied.

Our review builds on previous reviews [6,7] in several ways. We only included meta-epidemiological studies adopting a matched design, as these provide the most reliable evidence of the influence of reported study design characteristics on intervention effects [10]. We included 10 meta-epidemiological studies that were not included in the two previous reviews [32–35,37,39–41,46,48]. Rather than presenting only the average bias estimate of each meta-epidemiological study (as was done in [6,7]), which can be difficult for readers to interpret, we synthesised the average bias estimates for eight characteristics in random-effects meta-analyses. We concur with the previous AHRQ review [6] that lack of outcome assessor blinding and double blinding may exaggerate intervention effect estimates, yet we derived a more precise estimate of the influence of inadequate sequence generation and allocation concealment than the previous investigators. Ours is also the first systematic review to summarise estimates of between-trial heterogeneity associated with study characteristics, and variation in average bias across meta-analyses. The former was measured in only one meta-epidemiological study while the latter was measured in 11 (46%) meta-epidemiological studies. This low frequency is a shame because both features provide valuable data on whether certain methodological characteristics lead not only to bias, but also to more variation in trial effect estimates, and whether the average bias estimates are consistent across meta-analyses regardless of clinical area/intervention/type of outcome.

Our review has some limitations. We only considered methodological characteristics implied by the conceptual framework underlying the current Cochrane risk of bias tool for randomized trials, because it is unclear whether other characteristics investigated in meta-epidemiological studies (e.g. single-versus multi-centre status, early stopping) represent a specific bias, small-study effects, or spurious findings [54]. We relied on the existing AHRQ review by Berkman et al. [6] to identify meta-epidemiological studies published before 2012, rather than performing our own systematic search. Their search strategy was comprehensive, so we believe it is unlikely that we have missed earlier meta-epidemiological studies. We did not contact the authors of the included meta-epidemiological studies for a list of the meta-analyses/trials examined in their study, so cannot determine the number of overlapping meta-analyses/trials included in our analyses. However, the eligibility criteria described by the authors suggests that the included meta-epidemiological studies examined meta-analyses/trials conducted in clinical areas and published in years that differed from one another, and that differed from those included in the BRANDO study, which ensured no overlap between its constituent meta-epidemiological studies. Therefore, we believe that the frequency of overlapping meta-analyses/trials in our meta-analyses is likely to be small.

There are also important limitations of the included meta-epidemiological studies. Many meta-epidemiological studies examined a small number of meta-analyses, and so may have had insufficient power to reliably estimate associations [55]. Estimates of average bias due to one characteristic (e.g. allocation concealment) may be confounded by differences in other characteristics (e.g. lack of blinded participants, sample size). Few meta-epidemiological studies adjusted for confounders or adopted a within-trial design which reduced potential for confounding (e.g. [11]). Assessment of characteristics is often entirely based on what is reported in papers, and reported methods do not always reflect actual conduct [56,57]. Therefore, it remains unclear whether inadequate methods truly cause bias in intervention effect estimates or are an artefact of incomplete reporting of trials or confounding (or both). To improve the evidence base, future meta-epidemiological studies should report both univariable and multivariable analyses that adjust for potential confounders and, where available, assess risk of bias based on the more detailed methods that are often reported in trial protocols as well as methods reported in publications [58].

We encourage decision makers and systematic reviewers who rely on the results of randomized trials to routinely consider the risk of biases associated with the methods used. Our review suggests that particular caution is needed when interpreting the results of trials in which sequence generation, allocation concealment and blinding are not reported, and when outcome measures are subjectively assessed. This evidence is currently being taken into consideration in our work on a revision of the Cochrane risk of bias tool for randomized trials, which will include a new structure and clearer guidance that we anticipate will lead to more robust assessments.

Novel approaches are needed to examine the influence of attrition and selective reporting. Most previous meta-epidemiological studies of the influence of attrition have dichotomised trials based on some arbitrary amount of missing data (e.g. >20%). It would be more useful to know whether average bias varies according to different amounts of and reasons for missing data. Further, in previous meta-epidemiological studies of selective reporting, the authors only examined whether omission or addition of *any* trial outcome between the methods and results section biases the result for the *primary* outcome of the review. This approach is based on an assumption that selective reporting of *any* outcome leads to biased effect estimates for *all* outcomes. It is more informative to know whether the specific trial effect estimates that are assumed/known to have been selectively reported (e.g. because post-hoc, questionable analysis methods were used) are systematically different from trial effect estimates assumed/known to have not been selectively reported. No such investigation was conducted in any of the meta-epidemiological studies included in our review.

In conclusion, empirical evidence suggests that the following characteristics of randomized trials are associated with exaggerated intervention effect estimates: inadequate/unclear (versus adequate) sequence generation and allocation concealment, and no/unclear blinding of participants, blinding of outcome assessors and double blinding. The average bias appears to be greatest in trials of subjective outcomes. More research on the influence of attrition and biased reporting of results is needed. The development of novel methodological approaches for the empirical investigation of study design biases would also be valuable.

## Supporting Information

S1 PRISMA Checklist PRISMA Checklist. (DOC)

S1 Appendix Study protocol, search strategy, data collection form, and supplementary tables. (DOCX)
