# Supplementary material for: Empirical Evidence of Study Design Biases in Randomized Trials: Systematic Review of Meta-Epidemiological Studies
Source: PLoS One. 2016 Jul 11;11(7):e0159267. doi: 10.1371/journal.pone.0159267 (PMC4939945; doi:10.1371/journal.pone.0159267)
Supplement: S1 Appendix — (DOCX) [file pone.0159267.s002.docx]

**Supporting Information**

**Contents**

Appendix 1. Systematic review protocol…………………………………………………………….2

Appendix 2. Search strategies……………………………………………………………...……….10

Appendix 3. Data extraction items………………………………………………………….………12

Appendix 4. List of excluded studies………………………………………………………….……14

Appendix 5. Supplementary tables…………………………………………………………...……..25

Table S1. Clinical characteristics of included studies…………………………………...…...……..25

Table S2. Methodological characteristics of included studies……………………………...………31

Table S3. Study design characteristics examined in included studies………………………….......34

Table S4. Definitions of adequate, unclear and inadequate sequence generation……………….....36

Table S5. Definitions of adequate, unclear and inadequate allocation concealment.........................38

Table S6. Definitions of adequate, unclear and inadequate baseline imbalance…………………....41

Table S7. Definitions of adequate, unclear and inadequate blinding of participants…………….....42

Table S8. Definitions of adequate, unclear and inadequate blinding of personnel ……………..….43

Table S9. Definitions of adequate, unclear and inadequate blinding of outcome assessors............. 44

Table S10. Definitions of adequate, unclear and inadequate double blinding……………..……….45

Table S11. Definitions of adequate, unclear and inadequate attrition…………………...………….47

Table S12. Definitions of adequate, unclear and inadequate (selective) reporting……………..…..48

Table S13. Definitions of adequate, unclear and inadequate study design characteristics not classified elsewhere………………………………………………………………………………….………...49

Table S14. Average bias and heterogeneity associated with methodological characteristics, sub-grouped by type of intervention………………………………………………………………..…...50

**Appendix 1: Systematic review protocol**

**Empirical evidence of study design biases in randomized trials: systematic review protocol**

**Background**

The randomized controlled trial (RCT) is generally considered to produce the most credible estimates of the effects of interventions (1). For this reason, RCTs are often used to inform health care and policy decisions, either directly or via their inclusion in evidence syntheses. However, intervention effect estimates in RCTs can sometimes be biased, creating the potential for ineffective and harmful interventions being implemented into practice, and effective interventions not (2). Therefore, assessment of the risk of bias in RCTs is advised when interpreting the results.

The Cochrane risk of bias tool (3) was developed to provide a standardised approach for risk of bias assessment. The most recent version (released in 2011) includes six evidence-based domains (the type of bias addressed by each domain is presented in parentheses): random sequence generation (selection bias), allocation concealment (selection bias), blinding of participants and personnel (performance bias), blinding of outcome assessment (detection bias), incomplete outcome data (attrition bias), and selective reporting (reporting bias). An “Other bias” domain is also available to record additional threats to internal validity (e.g. carryover effect in cross-over RCTs). The tool has had widespread uptake from the systematic review community; its use is mandatory in Cochrane reviews, and it was the most commonly used tool in non-Cochrane reviews of RCTs published in 2012 (4). However, the tool requires updating to address several problems identified in previous evaluations, for example, its modest inter-rater reliability (5-7).

A revision of the Cochrane risk of bias tool should be informed by the most up-to-date empirical evidence of bias in RCTs. The most comprehensive attempt to identify such evidence is an Agency for Healthcare Research and Quality (AHRQ) report published in 2014 (8). The report summarised the results of 38 studies and concluded that there was empirical evidence of bias in relation to some aspects of trial conduct, but that estimates of the magnitude of bias were mostly imprecise. However, the AHRQ report is limited to studies published before September 2012, so a systematic review that includes more recent evidence is needed.

The aim of this systematic review was to synthesise the results of empirical studies that have investigated specific methodological characteristics that are associated with biased intervention effect estimates in RCTs.

**Eligibility criteria**

*Types of studies:* We will include meta-epidemiological studies investigating the association between specific methodological characteristics and intervention effect estimates in RCTs. We will only consider meta-epidemiological studies adopting a paired design. Pairing is most often done at the meta-analysis level, where a cohort of meta-analyses is assembled and the individual studies within each meta-analysis are classified into those with or without a particular methodological characteristic (such as adequate versus inadequate allocation concealment). Pairing can also be done at the trial level, where a cohort of trials is assembled and different measures of the same outcome in each trial, or sub-studies within each trial, are classified into those with or without a characteristic (such as blinded versus unblinded assessment of the same outcome) (9, 10). We will include meta-epidemiological studies regardless of the:

- sampling frame from which meta-analyses/trials were drawn (e.g. random sample of meta-analyses indexed in 2010 or all Cochrane reviews published in May 2012);
- clinical condition(s) investigated;
- type of intervention (e.g. pharmacological or non-pharmacological);
- type of outcome (e.g. objectively or subjectively assessed);
- type of outcome measure (e.g. dichotomous or continuous);
- type of effect measure (e.g. odds ratios (OR) or standardised mean difference (SMD));
- methods used to analyse the association between methodological characteristics and intervention effect estimates, and;
- variables used in the adjustment of associations (e.g. sample size, other source of bias, clinical condition).

We will exclude single systematic reviews and meta-analyses of RCTs which present a subgroup or sensitivity analysis based on a particular source of bias. We will also exclude studies that used a parallel design, where a cohort of RCTs was assembled (e.g. all child health related RCTs published in 2012), and meta-regression was performed to examine the relationship between a source of bias and trial effect estimates. Such studies do not control for the different outcomes measured across the trials, and so are at a higher risk of bias due to confounding. We will also exclude meta-epidemiological studies comparing randomized versus non-randomized studies investigating the same question.

*Types of methodological features:* We will only include studies investigating methodological features that can lead to the biases currently addressed by the Cochrane risk of bias tool for RCTs (see Table 1). We will include studies regardless of how the sources of bias were assessed/defined by the study authors. For example, older studies may have used the Jadad scale (11) to assess allocation concealment while more recent studies may have used the Cochrane risk of bias tool (3). Further, some studies may have categorised RCTs based on whether “double” or “single” or no blinding was performed, while other studies may have assessed which parties (i.e. patients, personnel) specifically were blinded. We will exclude studies investigating only the association between other characteristics and intervention effect estimates in RCTs (e.g. industry sponsorship (12), sample size (13), single versus multi-centre status (14, 15), stopping trials early for benefit or harm (16), and country of enrolment (17)).

**Table 1. Eligible sources of bias in randomized trials**

| **Type of bias** | **Possible methodological features that can lead to bias** |
| --- | --- |
| A. Bias arising from the randomisation process | 1. Inadequate generation of a random sequence 2. Inadequate allocation concealment 3. Imbalance in baseline characteristics 4. No adjustment for confounding in the analysis |
| B. Bias due to departures from intended interventions | 1. Non-blinded participants 2. Non-blinded clinician/provider 3. Unbalanced delivery of additional interventions or co-interventions 4. Participants switching interventions within the trial and being analysed in a group different from the one to which they were randomized |
| C. Bias due to missing/incomplete outcome data | 1. Missing/incomplete outcome data |
| D. Bias in measurement of outcomes | 1. Non-blinded outcome assessor 2. Non-blinded data analyst 3. Use of faulty measurement instruments (with low validity and reliability) |
| E. Bias in selection of the reported result | 1. Selective reporting of a subset of outcome domains, or of a subset of outcome measures or analyses for a particular outcome domain. |

*Types of outcomes:* Our primary outcomes are the association between each methodological characteristic and the (1) magnitude of the trial effect estimate (average bias), (2) increase in between-trial heterogeneity, and (3) heterogeneity in average bias, overall (i.e. regardless of the type of outcome or intervention). Secondary outcomes include the three associations above stratified by type of outcome (e.g. “mortality” versus “other objective” versus “subjective”) and type of intervention (e.g. “pharmacological” versus “non-pharmacological”), however defined by the study authors. We will include studies which present at least one of these estimates.

**Search strategy**

We will retrieve all meta-epidemiological studies included in the AHRQ report, which searched for studies published up to September 2012 (8). To identify more recent studies, we will search Ovid MEDLINE (Jan 2012 to May 2015) and Ovid EMBASE (Jan 2012 to May 2015). We will also search the Cochrane Database of Systematic Reviews for all reviews edited by the Methodology Review Group (on 20 May 2015), and abstract books of the 2011-2014 Cochrane Colloquia (available at <http://abstracts.cochrane.org/>) and of the 2011 and 2013 Clinical Trials Methodology Conference (available at <http://www.trialsjournal.com/supplements/12/S1/all> and <http://www.trialsjournal.com/supplements/14/S1/all>). We will review the reference lists of all included studies to identify additional studies. We will also review the list of studies included in two other relevant reviews (18, 19).

**Study selection**

One reviewer will screen all titles and abstracts retrieved from the searches. Two reviewers will independently screen all full text articles retrieved. Any disagreements regarding study eligibility will be resolved via discussion

**Data extraction and management**

One reviewer will extract data using a form developed in Microsoft Excel. A second reviewer will verify the accuracy of all average bias and heterogeneity effect estimates and confidence limits extracted.

The following data will be extracted:

- study characteristics, including the methodological characteristics investigated, how the characteristic was assessed (i.e. number of authors involved in assessment, inter-rater reliability of assessment), definitions of adequate/inadequate characteristics, number of included meta-analyses, number of RCTs included in the meta-analyses, sampling frame, areas of health care addressed, and range of years of publication of the meta-analyses;
- types of outcomes, interventions and comparators examined in the meta-analyses (which will be categorised using the classification systems described by Savovic et al. (20), when sufficient information about each is provided);
- effect estimates and measures of precision (e.g. ratio of odds ratio (ROR) and 95% confidence interval (95% CI);
- any confounding variables assessed by the study authors (e.g. sample size, other methodological characteristics);
- any methods used to deal with potential overlap of RCTs across the meta-analyses.

**Statistical analyses**

Characteristics of included studies will be summarised using frequencies and percentages for binary variables and medians and interquartile ranges (IQRs) for continuous variables.

We will analyse the association between a methodological characteristic and the magnitude of an intervention effect estimate (average bias) using the ratio of odds ratios (ROR), ratio of hazard ratios (RHR), or difference in standardised mean differences (dSMD) effect measure, whichever is reported by the study investigators. We will analyse the association between a methodological characteristic and between-trial heterogeneity, and the variation in average bias, using the standard deviation of underlying effects (tau) or I^2^. Only associations for individual characteristics will be analysed (i.e. we will not consider average bias in trials with both adequate allocation concealment *and* double blinding, or in trials rated at “overall high risk of bias”).

We anticipate that studies will vary in both their classification of the methodological characteristics of RCTs and the analysis model used to estimate average bias. For this reason, we will combine studies in a random-effects meta-analysis. We will use DerSimonian and Laird’s method of moments estimator to estimate the between-study variance (21). Statistical inconsistency will be quantified using the I^2^ statistic (22). We will not perform a meta-analysis if we suspect that the studies include many overlapping meta-analyses/trials (e.g. based on the publication year and topic areas of the meta-analyses), and in cases where the definitions of methodological characteristics are non-comparable between studies. The direction of effect will be standardised so that a ROR < 1, RHR <1 and dSMD <0 denotes a larger intervention effect estimate in trials with an inadequate/unclear characteristic.

**References**

1. OCEBM Levels of Evidence Working Group. "The Oxford 2011 Levels of Evidence". Oxford Centre for Evidence-Based Medicine. http://www.cebm.net/index.aspx?o=5653.

2. Ioannidis JPA, Greenland S, Hlatky MA, Khoury MJ, Macleod MR, Moher D, et al. Increasing value and reducing waste in research design, conduct, and analysis. The Lancet 2014;383(9912):166-75.

3. Higgins JPT, Altman DG, Gøtzsche PC, Jüni P, Moher D, Oxman AD, et al. The Cochrane Collaboration’s tool for assessing risk of bias in randomised trials. BMJ 2011;343:d5928.

4. Hopewell S, Boutron I, Altman DG, Ravaud P. Incorporation of assessments of risk of bias of primary studies in systematic reviews of randomised trials: a cross-sectional study. BMJ Open 2013;3:e003342.

5. Savovic J, Weeks L, Sterne JA, Turner L, Altman DG, Moher D, et al. Evaluation of the Cochrane Collaboration's tool for assessing the risk of bias in randomized trials: focus groups, online survey, proposed recommendations and their implementation. Systematic Reviews 2014;3:37.

6. Armijo-Olivo S, Ospina M, da Costa BR, Egger M, Saltaji H, Fuentes J, et al. Poor reliability between Cochrane reviewers and blinded external reviewers when applying the Cochrane risk of bias tool in physical therapy trials. PloS One 2014;9(5):e96920.

7. Hartling L, Hamm MP, Milne A, Vandermeer B, Santaguida PL, Ansari M, et al. Testing the risk of bias tool showed low reliability between individual reviewers and across consensus assessments of reviewer pairs. Journal of Clinical Epidemiology 2013;66(9):973-81.

8. Berkman ND, Santaguida PL, Viswanathan M, Morton SC. AHRQ Methods for Effective Health Care. The Empirical Evidence of Bias in Trials Measuring Treatment Differences. Rockville (MD): Agency for Healthcare Research and Quality (US); 2014.

9. Sterne JA, Juni P, Schulz KF, Altman DG, Bartlett C, Egger M. Statistical methods for assessing the influence of study characteristics on treatment effects in 'meta-epidemiological' research. Statistics in Medicine 2002;21(11):1513-24.

10. Savovic J, Jones H, Altman D, Harris R, Juni P, Pildal J, et al. Influence of reported study design characteristics on intervention effect estimates from randomised controlled trials: combined analysis of meta-epidemiological studies. Health Technology Assessment 2012;16(35):1-82.

11. Jadad AR, Moore RA, Carroll D, Jenkinson C, Reynolds DJM, Gavaghan DJ, et al. Assessing the quality of reports of randomized clinical trials: Is blinding necessary? Controlled Clinical Trials 1996;17(1):1-12.

12. Lundh A, Sismondo S, Lexchin J, Busuioc OA, Bero L. Industry sponsorship and research outcome. Cochrane Database of Systematic Reviews 2012;12:MR000033.

13. Dechartres A, Trinquart L, Boutron I, Ravaud P. Influence of trial sample size on treatment effect estimates: meta-epidemiological study. BMJ 2013;346:f2304.

14. Bafeta A, Dechartres A, Trinquart L, Yavchitz A, Boutron I, Ravaud P. Impact of single centre status on estimates of intervention effects in trials with continuous outcomes: meta-epidemiological study. BMJ 2012;344:e813.

15. Dechartres A, Boutron I, Trinquart L, Charles P, Ravaud P. Single-center trials show larger treatment effects than multicenter trials: evidence from a meta-epidemiologic study. Annals of Internal Medicine 2011;155(1):39-51.

16. Bassler D, Briel M, Montori VM, Lane M, Glasziou P, Zhou Q, et al. Stopping randomized trials early for benefit and estimation of treatment effects: systematic review and meta-regression analysis. JAMA 2010;303(12):1180-7.

17. Panagiotou OA, Contopoulos-Ioannidis DG, Ioannidis JP. Comparative effect sizes in randomised trials from less developed and more developed countries: meta-epidemiological assessment. BMJ 2013;346:f707.

18. Jacobs WC, Kruyt MC, Moojen WA, Verbout AJ, Oner FC. No evidence for intervention-dependent influence of methodological features on treatment effect. Journal of Clinical Epidemiology 2013;66(12):1347-55.e3.

19. Mills EJ, Ayers D, Chou R, Thorlund K. Are current standards of reporting quality for clinical trials sufficient in addressing important sources of bias? Contemporary Clinical Trials 2015;45(Pt A):2-7.

20. Savovic J, Jones HE, Altman DG, Harris RJ, Juni P, Pildal J, et al. Influence of reported study design characteristics on intervention effect estimates from randomized, controlled trials. Annals of Internal Medicine 2012;157(6):429-38.

21. DerSimonian R, Laird N. Meta-analysis in clinical trials. Controlled Clinical Trials 1986;7(3):177-88.

22. Higgins JP, Thompson SG, Deeks JJ, Altman DG. Measuring inconsistency in meta-analyses. BMJ 2003;327(7414):557-60.

**Appendix 2: Search strategies**

**Database: Ovid MEDLINE(R) 1946 to 25/05/2015**

Search Strategy:

--------------------------------------------------------------------------------

1. selection bias*.tw. (5287)
2. performance bias*.tw. (113)
3. detection bias*.tw. (409)
4. attrition bias*.tw. (193)
5. reporting bias*.tw. (775)
6. publication bias*.tw. (4176)
7. ((observer or intra-observer or intraobserver or inter-observer or interobserver) adj (bias* or variation*)).tw. (2515)
8. exp “bias (epidemiology)”/ (53854)
9. or/1-8 (64298)
10. meta-analysis.pt,ti,ab,sh. (72290)
11. (meta anal$ or metaanal$).ti,ab,sh. (87299)
12. ((methodol$ or systematic$ or quantitativ$) adj3 (review$ or overview$ or survey$)).ti. (37880)
13. ((methodol$ or systematic$ or quantitativ$) adj3 (review$ or overview$ or survey$)).ab. (54297)
14. ((pool$ or combined or combining) adj (data or trials or studies or results)).ti,ab. (14032)
15. or/10-14 (142784)
16. review.pt,sh. (1955779)
17. 15 and 16 (75640)
18. (meta-meta-anal$ or meta-review$ or meta-epidemiologic$ or metaepidemiologic$).ti,ab. (111)
19. exp in vitro techniques/ (525592)
20. simulation/ or computer simulation/ (146068)
21. simulation.tw. (85984)
22. 20 or 21 (198358)
23. exp DNA/ (661608)
24. exp Genetics/ (187780)
25. (genom* or genetic).ti. (225166)
26. or/23-25 (975488)
27. 9 not (19 or 22 or 26) (61150)
28. 27 and 17 (2993)
29. 28 or 18 (3091)
30. limit 29 to yr="2012-2015" (1532)

**Database: Embase <1974 to 2015 May 20>**

Search Strategy:

--------------------------------------------------------------------------------

1. selection bias*.tw. (7910)
2. performance bias*.tw. (157)
3. detection bias*.tw. (491)
4. attrition bias*.tw. (214)
5. reporting bias*.tw. (1074)
6. publication bias*.tw. (6388)
7. ((observer or intra-observer or intraobserver or inter-observer or interobserver) adj (bias* or variation*)).tw. (3228)
8. exp “bias (epidemiology)”/ (19687)
9. or/1-8 (36730)
10. meta-analysis.pt,ti,ab,sh. (121853)
11. (meta anal$ or metaanal$).ti,ab,sh. (144341)
12. ((methodol$ or systematic$ or quantitativ$) adj3 (review$ or overview$ or survey$)).ti. (55916)
13. ((methodol$ or systematic$ or quantitativ$) adj3 (review$ or overview$ or survey$)).ab. (81094)
14. ((pool$ or combined or combining) adj (data or trials or studies or results)).ti,ab. (19743)
15. or/10-14 (226690)
16. review.pt,sh. (2150818)
17. 15 and 16 (83389)
18. (meta-meta-anal$ or meta-review$ or meta-epidemiologic$ or metaepidemiologic$).ti,ab. (153)
19. exp in vitro techniques/ (4340986)
20. simulation/ or computer simulation/ (182383)
21. simulation.tw. (123005)
22. 20 or 21 (240551)
23. exp DNA/ (759263)
24. exp Genetics/ (635893)
25. (genom* or genetic).ti. (279206)
26. or/23-25 (1450335)
27. 9 not (19 or 22 or 26) (33529)
28. 27 and 17 (1986)
29. 28 or 18 (2137)
30. limit 29 to yr="2012-2015" (1108)

**Cochrane Methodology Review Group reviews**

Searched <http://www.cochranelibrary.com/app/content/browse/page/?context=editorial-group/Methodology%20Review%20Group> on 26/5/2015 (with no date restriction).

This site lists all reviews managed by the Cochrane Methodology Review Group.

**Cochrane Colloquium abstracts**

Searched <http://abstracts.cochrane.org/> on 26/5/2015 using the following terms (with no date restriction):

1. meta-epidemiology OR meta-epidemiological

**Clinical Trials Methodology Conference abstracts**

Searched abstracts of the 2011 and 2013 Clinical Trials Methodology Conference (available at <http://www.trialsjournal.com/supplements/12/S1/all> and <http://www.trialsjournal.com/supplements/14/S1/all>)

**Appendix 3. Data extraction items**

*General characteristics*

- Study ID
- Citation
- Design (e.g. collection of meta-analyses of trials with or without characteristic)
- Methodological feature(s) investigated (e.g. allocation concealment, double blinding)
- Areas of health care (% meta-analyses)
- Intervention types (% meta-analyses)
- Comparison types (% meta-analyses)
- Outcomes examined (% meta-analyses)
- Outcome types, e.g. binary (% meta-analyses)
- Sampling frame
- Publication years of meta-analyses
- Publication years of trials
- Risk of bias assessment method
- Inter-rater reliability for risk of bias assessment
- Total number of included meta-analyses
- Total number of included trials
- Median (IQR, range) sample size of meta-analyses
- Median (IQR, range) sample size of trials
- Analysis approach used (e.g. two-step meta-epidemiological (meta-meta-analytic) approach)
- Statistical analysis methods (details)
- How non-independence of data was addressed
- Supporting evidence regarding non-independence

*Association between a particular methodological characteristic and average bias and heterogeneity (separate forms per characteristic)*

- Study ID
- Definition of adequate (or low risk) characteristic
- Definition of unclear (or unclear risk) characteristic
- Definition of inadequate (or high risk) characteristic
- Comparison (e.g. High/unclear risk of bias versus low risk)
- Adjustment variables
- Outcome category (e.g. all, subjective)
- Binary, Continuous, Other outcome?
- Number of included meta-analyses
- Number of included trials
- Effect measure for average bias (e.g. ratio of odds ratios, difference of standardised mean differences)
- Direction of effect for average bias (e.g. ROR <1 = larger effect in high risk trials)
- Estimate of average bias
- Standard error of average bias
- Lower 95% confidence limit for average bias
- Upper 95% confidence limit for average bias
- Effect measure for increase in between-trial heterogeneity (e.g. increase in between-trial standard deviation)
- Estimate of increase in between-trial heterogeneity
- Standard error of increase in between-trial heterogeneity
- Lower 95% confidence limit for increase in between-trial heterogeneity
- Upper 95% confidence limit for increase in between-trial heterogeneity
- Effect measure for heterogeneity in average bias (e.g. between-meta-analysis standard deviation)
- Estimate of heterogeneity in average bias
- Standard error of heterogeneity in average bias
- Lower 95% confidence limit for heterogeneity in average bias
- Upper 95% confidence limit for heterogeneity in average bias

**Appendix 4. List of excluded studies**

| **Study ID** | **Reason for exclusion** |
| --- | --- |
| Ainsworth 2015 (1) | Not a meta-epidemiological study: case study of blinded versus unblinded assessment of the same outcome in a single RCT |
| Als-Nielsen 2003 (2) | Ineligible study design/conduct feature: association between funding/sponsorship and trial effect estimates |
| Armijo-Olivo 2015 (3) | Not a meta-epidemiological study: comparison of effect estimates of trials rated at high quality using different criteria (PEDro and Cochrane risk of bias tool) rather than comparison of low and high quality trials |
| Avni 2014 (4) | Not a meta-epidemiological study: meta-regression in a single systematic review of RCTs |
| Bafeta 2012 (5) | Ineligible study design/conduct feature: association between single versus multi-centre status and trial effect estimates |
| Bausell 2004 (6) | Not a meta-epidemiological study: comparison of trial with largest and smallest effect estimate in each meta-analysis |
| Bes-Rastrollo 2013 (7) | Ineligible study design/conduct feature: association between funding/sponsorship and trial effect estimates |
| Bohlius 2014 (8) | Not a meta-epidemiological study: single systematic review that included both published and unpublished trials; no bias assessment |
| Chan 2004 (9) | Not a meta-epidemiological study: investigation of selective non-reporting of outcomes in a cohort of trials |
| Chan 2004 (10) | Not a meta-epidemiological study: investigation of selective non-reporting of outcomes in a cohort of trials |
| Ciani 2013 (11) | Ineligible study design/conduct feature: association between surrogate versus final patient relevant outcomes and trial effect estimates |
| Clifford 2002 (12) | Not a meta-epidemiological study: bias assessment in a cohort of trials (parallel design) |
| Derry 2006 (13) | Not a meta-epidemiological study: sensitivity analysis of 35 acupuncture systematic reviews by removing trials without randomisation or double blinding |
| Døssing 2014a (14) | Awaiting assessment: data presented in a conference abstract |
| Døssing 2014b (15) | Awaiting assessment: data presented in a conference abstract |
| Dossing 2014c (16) | Awaiting assessment: data presented in a conference abstract |
| Dwan 2010 (17) | Not a meta-epidemiological study: investigation of selective non-reporting of outcomes in a cohort of trials |
| Egan 2012 (18) | Not a meta-epidemiological study: investigation of selective non-reporting of outcomes in a cohort of trials |
| Eriksen 2014 (19) | Not a meta-epidemiological study: meta-regression in a single systematic review of RCTs |
| Fenwick 2008 (20) | Not a meta-epidemiological study: meta-regression in a single systematic review of RCTs |
| Furlan 2008 (21) | Not a meta-epidemiological study: comparison of RCTs and non-randomized studies for the same question |
| Furukawa 2007 (22) | Not a meta-epidemiological study: investigation of selective non-reporting of outcomes in a cohort of trials |
| Gellatly 2007 (23) | Not a meta-epidemiological study: meta-regression in a single systematic of RCTs |
| Goto 2013 (24) | Not a meta-epidemiological study: sensitivity analyses based on risk of bias criteria in a single meta-analysis |
| Grimm 2013 (25) | Not a meta-epidemiological study: sensitivity analyses based on risk of bias criteria in a single meta-analysis |
| Hamm 2010 (26) | Not a meta-epidemiological study: bias assessment in a cohort of trials (parallel design) |
| Hartling 2011 (27) | Not a meta-epidemiological study: meta-regression in a single systematic review of RCTs |
| Hartling 2012 (28) | Not a meta-epidemiological study: meta-regression in a single systematic review of RCTs |
| Hartling 2009 (29) | Not a meta-epidemiological study: meta-regression in a single systematic review of RCTs |
| Hebert-Davies 2012 (30) | Not a meta-epidemiological study: systematic review of hip fracture trials and analysis of whether dementia patients were systematically excluded from the trials |
| Hempel 2012 (31) | Not a meta-epidemiological study: combination of 4 previous studies, analysed using meta-regression |
| Hempel 2013 (32) | Not a meta-epidemiological study: simulation study of power to detect trial quality moderator effects in meta-analyses (for use when planning meta-regressions) |
| Hempel 2011 (33) | Not a meta-epidemiological study: bias assessment in a cohort of trials (parallel design) |
| Herbison 2006 (34) | Not a meta-epidemiological study: comparison of different quality scales in terms of overlap of “high” versus “low” quality studies detected across the scales |
| Inaba 2009 (35) | Not a meta-epidemiological study: meta-regression in a single systematic review of RCTs |
| Jacobs 2011 (36) | Not a meta-epidemiological study: bias assessment in a cohort of trials (parallel design). Also systematic reviews included RCTs and non-randomized studies (secondary publication of Jacobs 2012) |
| Jacobs 2012 (37) | Not a meta-epidemiological study: bias assessment in a cohort of trials (parallel design). Also systematic reviews included both RCTs and non-randomized studies |
| Jacobs 2013 (38) | Not a meta-epidemiological study: systematic review and meta-analysis of studies investigating the association between study design characteristics and trial effect estimates |
| Jauhar 2014 (39) | Not a meta-epidemiological study: meta-regression in a single systematic review of RCTs |
| Juni 1999 (40) | Not a meta-epidemiological study: meta-regression in a single systematic review of RCTs |
| Kirkham 2010 (41) | Not a meta-epidemiological study: investigation of selective non-reporting of outcomes in a cohort of trials |
| Koletsi 2015 (42) | Not a meta-epidemiological study: bias assessment in a cohort of trials (parallel design) |
| Koretz 2014 (43) | Not a meta-epidemiological study: meta-regression in a single systematic review of RCTs |
| Kyzas 2005 (44) | Not a meta-epidemiological study and examines bias in prognostic studies |
| Lega 2013 (45) | Not a meta-epidemiological study: meta-regression in a single systematic review of RCTs |
| Linde 1999 (46) | Not a meta-epidemiological study: meta-regression in a single systematic review of RCTs |
| Liu 2011 (47) | Not a meta-epidemiological study: meta-regression in a single systematic review of RCTs |
| Lonjon 2014 (48) | Ineligible study design/conduct feature: meta-epidemiological study comparing RCTs versus non-randomized studies with propensity analysis |
| MacLehose 2000 (49) | Ineligible study design/conduct feature: comparison of RCTs and non-randomized studies for the same question |
| Maldini 2014 (50) | Not a meta-epidemiological study: comparison of prevalence studies using different recruitment methods |
| McKenzie 2011 (51) | Ineligible study design/conduct feature: association between final versus change from baseline values and trial effect estimates |
| McMahon 2008 (52) | Not a meta-epidemiological study: meta-regression in a single systematic review of RCTs |
| Mhaskar 2012 (53) | Not a meta-epidemiological study: bias assessment in a cohort of trials (parallel design) |
| Mohammed 2007 (54) | Not a meta-epidemiological study: single systematic review of RCTs |
| Moroz 2013 (55) | Not a meta-epidemiological study: systematic review of acupuncture trials that assessed how effective blinding was (by asking patients which treatment they believed they received) |
| Moustgaard 2014 (56) | Not a meta-epidemiological study: systematic review of methodology papers that provide definitions of “subjective” and “objective” outcomes, and audit of the use of these terms in clinical trial reports |
| Munder 2013 (57) | Ineligible study design/conduct feature: systematic review of empirical studies investigating “researcher allegiance bias” in psychotherapy research |
| Naci 2013 (58) | Not a meta-epidemiological study: single systematic review and meta-analysis of exercise versus drug trials |
| Nankervis 2012 (59) | Not a meta-epidemiological study: investigation of selective non-reporting of outcomes in a cohort of trials |
| Nuesch 2011 (60) | Ineligible study design/conduct feature: association between single- versus multi-centre status and trial effect estimates |
| Onishi 2014 (61) | Not a meta-epidemiological study: examination of how often statistically significant publication bias is present in reviews that do not assess for publication bias |
| Oomens 2013 (62) | Not a meta-epidemiological study: audit of the risk of bias in oral surgery trials |
| Panagiotou 2013 (63) | Ineligible study design/conduct feature: meta-epidemiological study of trials in less versus more developed countries |
| Papageorgiou 2014 (64) | Ineligible study design/conduct feature: meta-epidemiological study of studies indexed vs not in MEDLINE; English vs non-English studies; randomized vs non-randomized studies; small vs large RCTs; and trials with equal versus unequal arms |
| Papageorgiou 2015 (65) | Ineligible study design/conduct feature: comparison of statistical significance and heterogeneity of meta-analyses of binary outcomes when analysed using risk ratio, odds ratio or risk difference effect measure |
| Peura 2012 (66) | Ineligible study design/conduct feature: association between funding/sponsorship and trial effect estimates |
| Saini 2014 (67) | Not a meta-epidemiological study: investigation of selective non-reporting of outcomes in a cohort of trials |
| Saquib 2013 (68) | Ineligible study design/conduct feature: meta-epidemiological study comparing adjusted versus unadjusted effect estimates in RCTs |
| Savović 2010 (69) | Not a meta-epidemiological study: related publication of Savovic 2012 (methods used to develop the combined database; no results presented) |
| Seegers 2013 (70) | Ineligible study design/conduct feature: meta-epidemiological study comparing RCTs enrolling older adults only versus adults |
| Shang 2005 (71) | Not a meta-epidemiological study: bias assessment in a cohort of trials (parallel design) |
| Sichieri 2014 (72) | Ineligible study design/conduct feature: audit of how often cluster RCTs for obesity prevention adjust for baseline BMI in analyses |
| Smail-Faugeron 2014 (73) | Ineligible study design/conduct feature: meta-epidemiological study comparing split-mouth versus parallel-arm RCTs |
| Sterne 2002 (74) | Not a meta-epidemiological study: methodological paper describing the development of statistical methods for meta-epidemiological studies |
| Tarp 2013 (75) | Awaiting assessment: data presented in a conference abstract |
| Tierney 2005 (76) | Not a meta-epidemiological study: sensitivity analyses based on attrition in trials included in 14 IPD meta-analyses |
| Tonia 2013 (77) | Not a meta-epidemiological study: investigation of selective non-reporting of outcomes in a cohort of trials |
| Treadwell 2012 (78) | Not a meta-epidemiological study: AHRQ guidance for assessing equivalence and noninferiority |
| van Nieuwenhoven 2001 (79) | Not a meta-epidemiological study: meta-regression in a single systematic review of RCTs |
| van Tulder 2009 (80) | Not a meta-epidemiological study: bias assessment in a cohort of trials (parallel design) |
| Verhagen 2008 (81) | Not a meta-epidemiological study: meta-regression in a single systematic review of RCTs |
| Watzlawick 2014 (82) | Not a meta-epidemiological study: meta-regression in a single systematic review of RCTs |
| Wiedermann 2014 (83) | Not a meta-epidemiological study: investigation of selective non-reporting of outcomes in a cohort of trials |
| Worthen 2012 (84) | Not a meta-epidemiological study: single systematic review of RCTs with in-depth discussion of sources of risk of bias |
| Zhang 2012 (85) | Ineligible study design/conduct feature: meta-epidemiological study comparing small versus large RCTs |

**References**

1. Ainsworth H, Hewitt CE, Higgins S, Wiggins A, Torgerson DJ, Torgerson CJ. Sources of bias in outcome assessment in randomised controlled trials: a case study. Educational Research and Evaluation. 2015;21(1):3-14.

2. Als-Nielsen B, Chen W, Gluud C, Kjaergard LL. Association of funding and conclusions in randomized drug trials: a reflection of treatment effect or adverse events? JAMA. 2003;290(7):921-8.

3. Armijo-Olivo S, da Costa BR, Cummings GG, Ha C, Fuentes J, Saltaji H, et al. PEDro or Cochrane to Assess the Quality of Clinical Trials? A Meta-Epidemiological Study. PloS one. 2015;10(7):e0132634.

4. Avni T, Shiber-Ofer S, Leibovici L, Paul M. Assessment of bias in outcomes reported in trials on pneumonia: a systematic review. European journal of clinical microbiology & infectious diseases : official publication of the European Society of Clinical Microbiology. 2014;33(6):969-74.

5. Bafeta A, Dechartres A, Trinquart L, Yavchitz A, Boutron I, Ravaud P. Impact of single centre status on estimates of intervention effects in trials with continuous outcomes: meta-epidemiological study. BMJ. 2012;344:e813.

6. Bausell RB, Lee WL, Soeken KL, Li YF, Berman BM. Larger effect sizes were associated with higher quality ratings in complementary and alternative medicine randomized controlled trials. Journal of clinical epidemiology. 2004;57(5):438-46.

7. Bes-Rastrollo M, Schulze MB, Ruiz-Canela M, Martinez-Gonzalez MA. Financial conflicts of interest and reporting bias regarding the association between sugar-sweetened beverages and weight gain: a systematic review of systematic reviews. PLoS medicine. 2013;10(12):e1001578-e.

8. Bohlius J, Tonia T, Nuesch E, Juni P, Fey MF, Egger M, et al. Effects of erythropoiesis-stimulating agents on fatigue- and anaemia-related symptoms in cancer patients: systematic review and meta-analyses of published and unpublished data. British journal of cancer. 2014;111(1):33-45.

9. Chan AW, Hrobjartsson A, Haahr MT, Gotzsche PC, Altman DG. Empirical evidence for selective reporting of outcomes in randomized trials: comparison of protocols to published articles. Jama. 2004;291(20):2457-65.

10. Chan AW, Krleza-Jeric K, Schmid I, Altman DG. Outcome reporting bias in randomized trials funded by the Canadian Institutes of Health Research. Cmaj. 2004;171(7):735-40.

11. Ciani O, Buyse M, Garside R, Pavey T, Stein K, Sterne JAC, et al. Comparison of treatment effect sizes associated with surrogate and final patient relevant outcomes in randomised controlled trials: meta-epidemiological study. BMJ (Clinical research ed). 2013;346:f457.

12. Clifford TJ, Barrowman NJ, Moher D. Funding source, trial outcome and reporting quality: are they related? Results of a pilot study. BMC health services research. 2002;2(1):18.

13. Derry CJ, Derry S, McQuay HJ, Moore RA. Systematic review of systematic reviews of acupuncture published 1996-2005. Clinical medicine (London, England). 2006;6(4):381-6.

14. Døssing A, Tarp S, Furst D, Gluud C, Beyene J, Brandt Hansen B, et al. Interpreting trial results following use of different intention-to-treat approaches for preventing attrition bias: a meta-epidemiological study. In: Evidence-Informed Publich Health: Opportunities and Challenges. Abstracts of the 22nd Cochrane Colloquium; 2014 21-26 Sep; Hyderabad, India. John Wiley & Sons;. 2014.

15. Døssing A, Tarp S, Furst D, Gluud C, Beyene J, Brandt Hansen B, et al. How handling of missing data impacts trial results: a meta-epidemiological study. In: Evidence-Informed Publich Health: Opportunities and Challenges. Abstracts of the 22nd Cochrane Colloquium; 2014 21-26 Sep; Hyderabad, India. John Wiley & Sons. 2014.

16. Dossing A, Tarp S, Furst DE, Gluud C, Beyene J, Hansen BB, et al. Attrition bias in rheumatoid arthritis randomised trials with different modified intention-to-treat approaches: A meta-epidemiological study. Annals of the rheumatic diseases. 2014;73(Suppl 2):326.

17. Dwan K, Gamble C, Kolamunnage-Dona R, Mohammed S, Powell C, Williamson PR. Assessing the potential for outcome reporting bias in a review: a tutorial. Trials. 2010;11:52.

18. Egan G, Lee J, Minhas R, Tejani AM. Does outcome reporting bias "cause" cancer? Risks associated with hidden data on angiotensin receptor blockers. Can J Hosp Pharm. 2012;65(5):387-93.

19. Eriksen P, Bartels EM, Altman RD, Bliddal H, Juhl C, Christensen R. Risk of bias and brand explain the observed inconsistency in trials on glucosamine for symptomatic relief of osteoarthritis: a meta-analysis of placebo-controlled trials. Arthritis care & research. 2014;66(12):1844-55.

20. Fenwick J, Needleman IG, Moles DR. The effect of bias on the magnitude of clinical outcomes in periodontology: a pilot study. Journal of clinical periodontology. 2008;35(9):775-82.

21. Furlan AD, Tomlinson G, Jadad AA, Bombardier C. Examining heterogeneity in meta-analysis: comparing results of randomized trials and nonrandomized studies of interventions for low back pain. Spine. 2008;33(3):339-48.

22. Furukawa TA, Watanabe N, Omori IM, Montori VM, Guyatt GH. Association between unreported outcomes and effect size estimates in Cochrane meta-analyses. Jama. 2007;297(5):468-70.

23. Gellatly J, Bower P, Hennessy S, Richards D, Gilbody S, Lovell K. What makes self-help interventions effective in the management of depressive symptoms? Meta-analysis and meta-regression. Psychological medicine. 2007;37(9):1217-28.

24. Goto A, Arah OA, Goto M, Terauchi Y, Noda M. Severe hypoglycaemia and cardiovascular disease: systematic review and meta-analysis with bias analysis. BMJ (Clinical research ed). 2013;347:f4533.

25. Grimm NL, Shea KG, Leaver RW, Aoki SK, Carey JL. Efficacy and degree of bias in knee injury prevention studies: a systematic review of RCTs. Clinical orthopaedics and related research. 2013;471(1):308-16.

26. Hamm MP, Hartling L, Milne A, Tjosvold L, Vandermeer B, Thomson D, et al. A descriptive analysis of a representative sample of pediatric randomized controlled trials published in 2007. BMC pediatrics. 2010;10:96.

27. Hartling L, Bond K, Vandermeer B, Seida J, Dryden DM, Rowe BH. Applying the risk of bias tool in a systematic review of combination long-acting beta-agonists and inhaled corticosteroids for persistent asthma. PloS one. 2011;6(2):e17242.

28. Hartling L, Hamm M, Milne A, Vandermeer B, Santaguida PL, Ansari M, et al. AHRQ Methods for Effective Health Care. Validity and Inter-Rater Reliability Testing of Quality Assessment Instruments. Rockville (MD): Agency for Healthcare Research and Quality (US); 2012.

29. Hartling L, Ospina M, Liang Y, Dryden DM, Hooton N, Krebs Seida J, et al. Risk of bias versus quality assessment of randomised controlled trials: cross sectional study. Bmj. 2009;339:b4012.

30. Hebert-Davies J, Laflamme GY, Rouleau D, Health, investigators F. Bias towards dementia: are hip fracture trials excluding too many patients? A systematic review. Injury. 2012;43(12):1978-84.

31. Hempel S, Miles J, Suttorp MJ, Wang Z, Johnsen B, Morton S, et al. AHRQ Methods for Effective Health Care. Detection of Associations Between Trial Quality and Effect Sizes. Rockville (MD): Agency for Healthcare Research and Quality (US); 2012.

32. Hempel S, Miles JNV, Booth MJ, Wang Z, Morton SC, Shekelle PG. Risk of bias: a simulation study of power to detect study-level moderator effects in meta-analysis. Systematic reviews. 2013;2:107.

33. Hempel S, Suttorp MJ, Miles JNV, Wang Z, Maglione M, Morton S, et al. AHRQ Methods for Effective Health Care. Empirical Evidence of Associations Between Trial Quality and Effect Size. Rockville (MD): Agency for Healthcare Research and Quality (US); 2011.

34. Herbison P, Hay-Smith J, Gillespie WJ. Adjustment of meta-analyses on the basis of quality scores should be abandoned. Journal of clinical epidemiology. 2006;59(12):1249-56.

35. Inaba Y, Chen JA, Mehta N, Bergmann SR. Impact of single or multicentre study design on the results of trials examining the efficacy of adjunctive devices to prevent distal embolisation during acute myocardial infarction. EuroIntervention : journal of EuroPCR in collaboration with the Working Group on Interventional Cardiology of the European Society of Cardiology. 2009;5(3):375-83.

36. Jacobs W, Kruyt M, Verbout A, Oner C. Heterogeneity in systematic reviews on spinal surgery: A meta-epidemiological study. In: Abstracts of the 19th Cochrane Colloquium; 2011 19-22 Oct; Madrid, Spain. John Wiley & Sons; 2011.

37. Jacobs WCH, Kruyt MC, Verbout AJ, Oner FC. Effect of methodological quality measures in spinal surgery research: a metaepidemiological study. The spine journal : official journal of the North American Spine Society. 2012;12(4):339-48.

38. Jacobs WC, Kruyt MC, Moojen WA, Verbout AJ, Oner FC. No evidence for intervention-dependent influence of methodological features on treatment effect. Journal of clinical epidemiology. 2013;66(12):1347-55.e3.

39. Jauhar S, McKenna PJ, Radua J, Fung E, Salvador R, Laws KR. Cognitive-behavioural therapy for the symptoms of schizophrenia: systematic review and meta-analysis with examination of potential bias. The British journal of psychiatry : the journal of mental science. 2014;204(1):20-9.

40. Juni P, Witschi A, Bloch R, Egger M. The hazards of scoring the quality of clinical trials for meta-analysis. Jama. 1999;282(11):1054-60.

41. Kirkham JJ, Dwan KM, Altman DG, Gamble C, Dodd S, Smyth R, et al. The impact of outcome reporting bias in randomised controlled trials on a cohort of systematic reviews. Bmj. 2010;340:c365.

42. Koletsi D, Spineli LM, Lempesi E, Pandis N. Risk of bias and magnitude of effect in orthodontic randomized controlled trials: a meta-epidemiological review. European journal of orthodontics. 2015.

43. Koretz RL, Lipman TO. The presence and effect of bias in trials of early enteral nutrition in critical care. Clinical nutrition (Edinburgh, Scotland). 2014;33(2):240-5.

44. Kyzas PA, Loizou KT, Ioannidis JP. Selective reporting biases in cancer prognostic factor studies. Journal of the National Cancer Institute. 2005;97(14):1043-55.

45. Lega JC, Mismetti P, Cucherat M, Fassier T, Bertoletti L, Chapelle C, et al. Impact of double-blind vs. open study design on the observed treatment effects of new oral anticoagulants in atrial fibrillation: a meta-analysis. Journal of thrombosis and haemostasis : JTH. 2013;11(7):1240-50.

46. Linde K, Scholz M, Ramirez G, Clausius N, Melchart D, Jonas WB. Impact of study quality on outcome in placebo-controlled trials of homeopathy. Journal of clinical epidemiology. 1999;52(7):631-6.

47. Liu CJ, LaValley M, Latham NK. Do unblinded assessors bias muscle strength outcomes in randomized controlled trials of progressive resistance strength training in older adults? American journal of physical medicine & rehabilitation / Association of Academic Physiatrists. 2011;90(3):190-6.

48. Lonjon G, Boutron I, Trinquart L, Ahmad N, Aim F, Nizard R, et al. Comparison of treatment effect estimates from prospective nonrandomized studies with propensity score analysis and randomized controlled trials of surgical procedures. Annals of surgery. 2014;259(1):18-25.

49. MacLehose RR, Reeves BC, Harvey IM, Sheldon TA, Russell IT, Black AM. A systematic review of comparisons of effect sizes derived from randomised and non-randomised studies. Health technology assessment (Winchester, England). 2000;4(34):1-154.

50. Maldini C, La Valley MP, Druce K, Basu N, Mahr A. Analyzing the heterogeneity of behcet's disease prevalence: A meta-epidemiological approach. Annals of the rheumatic diseases. 2014;73(Suppl 2):547-8.

51. McKenzie J, Deeks J. Is it reasonable to pool estimates of intervention effect estimated from different analytical methods for continuous outcomes? A meta-epidemiological study. In: Abstracts of the 19th Cochrane Colloquium; 2011 19-22 Oct; Madrid, Spain. John Wiley & Sons; 2011.

52. McMahon B, Holly L, Harrington R, Roberts C, Green J. Do larger studies find smaller effects? The example of studies for the prevention of conduct disorder. European child & adolescent psychiatry. 2008;17(7):432-7.

53. Mhaskar R, Djulbegovic B, Magazin A, Soares HP, Kumar A. Published methodological quality of randomized controlled trials does not reflect the actual quality assessed in protocols. Journal of clinical epidemiology. 2012;65(6):602-9.

54. Mohammed S, Goodacre S. Intravenous and nebulised magnesium sulphate for acute asthma: systematic review and meta-analysis. Emergency medicine journal : EMJ. 2007;24(12):823-30.

55. Moroz A, Freed B, Tiedemann L, Bang H, Howell M, Park JJ. Blinding measured: A systematic review of randomized controlled trials of acupuncture. Evidence-Based Complementary and Alternative Medicine. 2013;2013:708251.

56. Moustgaard H, Bello S, Miller FG, Hrobjartsson A. Subjective and objective outcomes in randomized clinical trials: definitions differed in methods publications and were often absent from trial reports. Journal of clinical epidemiology. 2014;67(12):1327-34.

57. Munder T, Brutsch O, Leonhart R, Gerger H, Barth J. Researcher allegiance in psychotherapy outcome research: an overview of reviews. Clinical psychology review. 2013;33(4):501-11.

58. Naci H, Ioannidis JPA. Comparative effectiveness of exercise and drug interventions on mortality outcomes: metaepidemiological study. BMJ (Clinical research ed). 2013;347:f5577.

59. Nankervis H, Baibergenova A, Williams HC, Thomas KS. Prospective registration and outcome-reporting bias in randomized controlled trials of eczema treatments: a systematic review. The Journal of investigative dermatology. 2012;132(12):2727-34.

60. Nuesch E, Trelle S, Reichenbach S, Juni P. Overestimation of treatment benefits in single-centre osteoarthritis trials may be related to sample size: Meta-epidemiological study. In: Abstracts of the 19th Cochrane Colloquium; 2011 19-22 Oct; Madrid, Spain. John Wiley & Sons; 2011.

61. Onishi A, Furukawa TA. Publication bias is underreported in systematic reviews published in high-impact-factor journals: metaepidemiologic study. Journal of clinical epidemiology. 2014;67(12):1320-6.

62. Oomens MAEM, Heymans MW, Forouzanfar T. Risk of bias in research in oral and maxillofacial surgery. The British journal of oral & maxillofacial surgery. 2013;51(8):913-9.

63. Panagiotou OA, Contopoulos-Ioannidis DG, Ioannidis JP. Comparative effect sizes in randomised trials from less developed and more developed countries: meta-epidemiological assessment. BMJ. 2013;346:f707.

64. Papageorgiou SN, Antonoglou GN, Tsiranidou E, Jepsen S, Jager A. Bias and small-study effects influence treatment effect estimates: a meta-epidemiological study in oral medicine. Journal of clinical epidemiology. 2014;67(9):984-92.

65. Papageorgiou SN, Tsiranidou E, Antonoglou GN, Deschner J, Jager A. Choice of effect measure for meta-analyses of dichotomous outcomes influenced the identified heterogeneity and direction of small-study effects. Journal of clinical epidemiology. 2015;68:534-41.

66. Peura PK, Martikainen JA, Purmonen TT, Turunen JHO. Sponsorship-related outcome selection bias in published economic studies of triptans: systematic review. Medical decision making : an international journal of the Society for Medical Decision Making. 2012;32(2):237-45.

67. Saini P, Loke YK, Gamble C, Altman DG, Williamson PR, Kirkham JJ. Selective reporting bias of harm outcomes within studies: Findings from a cohort of systematic reviews. BMJ. 2014;349:g6501.

68. Saquib N, Saquib J, Ioannidis JPA. Practices and impact of primary outcome adjustment in randomized controlled trials: meta-epidemiologic study. BMJ (Clinical research ed). 2013;347:f4313.

69. Savović J, Harris RJ, Wood L, Beynon R, Altman D, Als-Nielsen B, et al. Development of a combined database for meta-epidemiological research. Research Synthesis Methods. 2010;1(3-4):212-25.

70. Seegers V, Trinquart L, Boutron I, Ravaud P. Comparison of treatment effect estimates for pharmacological randomized controlled trials enrolling older adults only and those including adults: a meta-epidemiological study. PloS one. 2013;8(5):e63677.

71. Shang A, Huwiler-Muntener K, Nartey L, Juni P, Dorig S, Sterne JA, et al. Are the clinical effects of homoeopathy placebo effects? Comparative study of placebo-controlled trials of homoeopathy and allopathy. Lancet. 2005;366(9487):726-32.

72. Sichieri R, Cunha DB. Unbalanced baseline in school-based interventions to prevent obesity: adjustment can lead to bias - a systematic review. Obesity facts. 2014;7(4):221-32.

73. Smail-Faugeron V, Fron-Chabouis H, Courson F, Durieux P. Comparison of intervention effects in split-mouth and parallel-arm randomized controlled trials: a meta-epidemiological study. BMC medical research methodology. 2014;14:64.

74. Sterne JA, Juni P, Schulz KF, Altman DG, Bartlett C, Egger M. Statistical methods for assessing the influence of study characteristics on treatment effects in 'meta-epidemiological' research. Stat Med. 2002;21(11):1513-24.

75. Tarp S, Amarilyo G, Woo JM, Li W, Bliddal H, Christensen R, et al. Agreements and discrepancies between the food and drug administration (FDA) reports and journal papers on biologic agents approved for rheumatoid arthritis: A meta-epidemiological study. Annals of the rheumatic diseases. 2013;72(Suppl 3):A773.

76. Tierney JF, Stewart LA. Investigating patient exclusion bias in meta-analysis. International journal of epidemiology. 2005;34(1):79-87.

77. Tonia T, Schwarzer G, Bohlius J. Cancer, meta-analysis and reporting biases: the case of erythropoiesis-stimulating agents. Swiss medical weekly. 2013;143:w13776.

78. Treadwell J, Uhl S, Tipton K, Singh S, Santaguida L, Sun X, et al. AHRQ Methods for Effective Health Care. Assessing Equivalence and Noninferiority. Rockville (MD): Agency for Healthcare Research and Quality (US); 2012.

79. van Nieuwenhoven CA, Buskens E, van Tiel FH, Bonten MJ. Relationship between methodological trial quality and the effects of selective digestive decontamination on pneumonia and mortality in critically ill patients. Jama. 2001;286(3):335-40.

80. van Tulder MW, Suttorp M, Morton S, Bouter LM, Shekelle P. Empirical evidence of an association between internal validity and effect size in randomized controlled trials of low-back pain. Spine. 2009;34(16):1685-92.

81. Verhagen AP, de Vet HC, Willemsen S, Stijnen T. A meta-regression analysis shows no impact of design characteristics on outcome in trials on tension-type headaches. Journal of clinical epidemiology. 2008;61(8):813-8.

82. Watzlawick R, Sena ES, Dirnagl U, Brommer B, Kopp MA, Macleod MR, et al. Effect and reporting bias of RhoA/ROCK-blockade intervention on locomotor recovery after spinal cord injury: a systematic review and meta-analysis. JAMA neurology. 2014;71(1):91-9.

83. Wiedermann CJ. Reporting bias in trials of volume resuscitation with hydroxyethyl starch. Wiener klinische Wochenschrift. 2014;126(7-8):189-94.

84. Worthen J, Waterman BR, Davidson PA, Lubowitz JH. Limitations and sources of bias in clinical knee cartilage research. Arthroscopy : the journal of arthroscopic & related surgery : official publication of the Arthroscopy Association of North America and the International Arthroscopy Association. 2012;28(9):1315-25.

85. Zhang Z, Xu X, Ni H. Small studies may overestimate the effect sizes in critical care meta-analyses: A meta-epidemiological study. Critical Care. 2012;17:R2.

**Appendix 5: Supplementary tables**

**Table S1: Clinical characteristics of included studies**

| **Study ID** | **Sampling frame** | **Areas of health care (% meta-analyses)** | **Interventions (% meta-analyses)** | **Comparators (% meta-analyses)** | **Outcomes (% meta-analyses)** | **No. meta-analyses (trials)** | **Publication years of meta-analyses** | **Publication years of trials** |
| --- | --- | --- | --- | --- | --- | --- | --- | --- |
| Abraha 2015 | Random selection of 5% of intervention reviews published 2006-2010 with a meta-analysis that included at least one trial that deviated from the standard intention to treat approach | Varied (includes neoplasms, injections, mental health, CVD, pregnancy, neurological conditions) | Pharmacologic (100%) | Placebo (68%); Standard care (32%) | Objective (38%); Subjective (62%) | 50 (322) | Range 2006-2010 | Range 1970-2009 |
| Als-Nielsen 2004 | Random sample of Cochrane reviews published in Issue 2, 2001 that included ≥5 trials with binary outcomes | Varied (NR) | Pharmacologic; Procedural; Behavioural (% NR) | No treatment; Placebo; Active treatment (% NR) | NR | 48 (523) | 2001 | NR |
| Armijo-Olivo 2015 | Random sample of Cochrane meta-analyses of continuous outcomes published from 2005-2011, that included ≥3 RCTs where ≥1 of the interventions was physical therapy | Musculoskeletal (51%); Cardio-respiratory (21%); Neurological (14%); Other (14%) | Non-pharmacologic (physical therapy) (100%) | Placebo or standard care (100%) | Objective (60%); Subjective (40%) | 43 (393) | Range 2008-2011 | NR |
| Balk 2002 | Meta-analyses in 4 areas (CVD, infectious disease, pediatrics, surgery) indexed in MEDLINE (1966-2000) and CDSR (issue 4, 2000), which included at least 6 RCTs, examined binary outcomes and demonstrated significant between-trial heterogeneity | CVD (31%); Infectious disease (23%); Pediatrics (19%); Surgery (27%) | Pharmacologic; Surgery (% not reported) | Not reported | Mortality; Infections; Complications of surgery; Other (% NR) | 26 (276) | Range 1983-2000 | NR |
| Bialy 2014 | All reviews of surfactant, corticosteroids, indomethacin, ibuprofen, nitric oxide and head/total body cooling for infants in CDSR up to May 2010, with a meta-analysis of a binary outcome | Neonatal health (100%) | Pharmacologic (96%); Procedural (4%) | Active (30%); Inactive (61%); Both (9%) | Mortality (60%); Other (40%) | 23 (207) | Median 2007 (range 1997-2011) | Median 1997 (range 1972-2009) |
| BRANDO (Savović 2012) | Seven previously published meta-epidemiological studies (Als-Nielsen 2004, Balk 2004, Contopoulos-Ioannidis 2005, Egger 2003, Kjaergard 2001, Pildal 2007, Schulz 1995) | Pregnancy and childbirth (24%); Mental and behavioural (11%); Circulatory system (13%); Digestive system (7%); Respiratory system (6%); Other (39%) | Pharmacologic (69%); Surgical (6%); Psychosocial, behavioural or educational (6%); Other (19%) | Placebo or no treatment (74%); Standard care (7%); Active comparison (19%); Mixture (1%) | All-cause mortality (19%); Other objective (15%); Objectively measured but potentially influenced by clinician judgement (18%); Subjective (42%); Mixture (6%) | 234 (1973) | Median 2000 (IQR 2000-2001, range 1983-2005) | Median 1989 (IQR 1983-1994, range 1948-2002) |
| Chaimani 2013 | PubMed-indexed meta-analyses of RCTs published by the end of March 2011, in which ≥3 treatments were included and the data had been analysed with a valid statistical method for indirect comparisons or network meta-analysis | Varied (NR) | Varied (NR) | Placebo, no treatment or standard care (100%) | Mortality (33%); Other (67%) | 20 (377) | Range 1999-2011 | NR |
| Contopoulos-Ioannidis 2005 | Mental health-related interventions identified from the Mental Health Library, 2002 (Issue 1) | Mental health (100%) | Pharmacologic (31%); Non-pharmacologic (69%) | NR | Psychological symptoms (50%); Smoking cessation (44%); Dropouts (6%) | 16 (133) | Median 1997 (range 1995-2002) | NR |
| Egger 2003 | Meta-analyses published in the CDSR (Issue 1, 1998) that that had performed comprehensive literature searches and included ≥5 trials with binary outcomes | Infectious diseases; Neonatology; Neurology; Obstetrics & gynaecology; Psychiatry; Other (% NR) | Pharmacologic (82%); Non-pharmacologic (18%) | NR | NR | 45 (399) | 1998 | Range 1950-1998 |
| Hartling 2014 | Child-relevant intervention reviews in the CDSR, which included ≥5 RCTs involving only pediatric patients (ages 0 to 17 years). | Child health (100%) | Pharmacologic (41%); Non-pharmacologic (59%) | Placebo (41%); No intervention (12%); Active comparison (12%); Mixed (35%) | Objective (65%); Subjective (35%) | 17 (287) | NR | Median 1995 (range 1965-2010) |
| Herbison 2011 | Systematic reviews in the CDSR (issue 1, 2001) that included ≥10 trials with binary outcomes, at least one of which had >500 people randomized to each arm | Pregnancy and childbirth (50%); CVD (17%); Smoking cessation (11%); Other (22%) | Pharmacologic (78%); Procedural (22%) | NR | Objective (95%); Subjective (5%) | 65 (389) | 2001 | Not reported |
| Hrobjartsson 2012 | RCTs with blinded and non-blinded assessment of the same binary outcome, published up to Jan 2010 | General surgery (19%); Cosmetic surgery (19%); Orthopaedic surgery (14%); Other (48%) | Surgery or procedure (67%); Pharmacologic (24%); Other (9%) | Standard care (86%); Placebo (14%) | Subjective (100%) | (21)* | NA* | Range 1991-2011 |
| Hrobjartsson 2013 | RCTs with blinded and non-blinded assessment of the same binary outcome, published up to Jan 2010 | Neurology (25%); Cosmetic surgery (19%); CVD or Psychiatry or Otolaryngology (13% each); Other (19%) | Surgery or procedure (69%); Pharmacologic (31%) | Standard care (75%); No treatment or placebo (25%) | Subjective (100%) | (16)* | NA* | Range 1983-2010 |
| Hrobjartsson 2014a | RCTs with blinded and non-blinded assessment of the same binary outcome, published up to Sept 2013 | Opthamology (50%); Orthopaedic surgery (22%); Other (28%) | Pharmacologic (78%); Procedural (22%) | Standard care/active control (83%); No treatment or placebo (27%) | Subjective (100%) | (18)* | NA* | Range 1991-2011 |
| Hrobjartsson 2014b | Four- or three-armed clinical trials that randomized patients to a blinded sub-study and an otherwise identical nonblind sub-study, published up to March 2013 | Rheumatology (50%); Neurology (17%); Other (33%) | CAM pharmacologic (8%); CAM non-pharmacologic (92%) | Placebo or no treatment (100%) | Subjective (100%); Objective (17%) | (12)* | NA* | Range 2000-2011 |
| Kjaergard 2001 | Meta-analyses identified from CDSR or MEDLINE that included at least one large trial (≥1000 participants) | CVD (45%); Pregnancy and childbirth (36%); Other (18%) | Varied (NR) | NR | Mortality (36%); Other objective (64%) | 14 (190) | Median 1998 (range 1990-1998) | IQR 1986-1992, range 1960-1998 |
| Moher 1998 | Random sample of meta-analyses with binary outcomes, with no formal incorporation of quality scores in the quantitative analysis | Circulatory diseases (36%); Digestive diseases (27%); Mental health (27%); Pregnancy and childbirth (9%) | Varied (NR) | NR | Objective (68%); Subjective (32%) | 11 (127) | Range 1988-1995 | Range 1960-1995 |
| Nuesch 2009a | Meta-analyses of controlled trials for osteoarthritis pain indexed in CDSR, MEDLINE, EMBASE or CINAHL up to Nov 2007 | Osteoarthritis of the knee or hip (100%) | Pharmacologic (69%); Non-pharmacologic (31%) | Placebo, sham intervention or no intervention control (100%) | Subjective (patient-reported pain) (100%) | 16 (175) | Range 2003-2007 | Range 1980-2007 |
| Nuesch 2009b | Meta-analyses of controlled trials for osteoarthritis pain indexed in CDSR, MEDLINE, EMBASE or CINAHL up to Nov 2007 | Osteoarthritis of the knee or hip (100%) | Pharmacologic (57%); Non-pharmacologic (43%) | Placebo, sham intervention or no intervention control (100%) | Subjective (patient-reported pain) (100%) | 14 (167) | Range 2003-2007 | Range 1980-2007 |
| Papageorgiou 2015 | Systematic reviews in the field of oral medicine, with ≥1 meta-analysis of interventional studies with different designs, indexed up to July 2014 | Oral medicine (100%) | Procedural (100%) | No treatment or standard care (100%) | Objective (96%); Subjective (4%) | 25 (75) | Range 2010-2014 | NR |
| Pildal 2007 | Systematic reviews in the CDSR (Issue 2, 2003) or PubMed (2001-2002), with a binary outcome from a meta-analysis presented as the first statistically significant result that supported a conclusion in favour of one of the interventions | Varied (NR) | Varied (NR) | NR | NR | 29 (284) | Range 2001-2002 | NR |
| Schulz 1995 | Meta-analyses published by the Pregnancy and Childbirth Group of the Cochrane Collaboration, which included ≥5 trials with ≥1 with adequate allocation concealment and one without. | Pregnancy and childbirth (100%) | Varied (NR) | NR | NR | 33 (250) | Range 1989-1993 | Range 1955-1992 |
| Unverzagt 2013 | Systematic reviews in the CDSR (issue 11, 2011) on patients with cardiogenic shock, severe sepsis, and septic shock, with a meta-analysis of all-cause mortality including ≥3 RCTs | Critical care medicine (100%) | Pharmacologic (83%); Procedural (17%) | Placebo or standard care (83%); Other active intervention (17%) | Mortality (100%) | 12 (82) | Range 2002-2011 | Range 1956-2010 |
| Wood 2008 | Three previously published meta-epidemiological studies (Egger 2003, Kjaergard 2001, Schulz 1995) | Varied (NR) | Pharmacologic (61%); Prevention or screening (15%); Other (24%) | NR | Objective (53%); Subjective (47%) | 146 (1346) | Median 1998 (range 1990-2000) | Median (1987 (range 1962-1998) |

*No meta-analyses because outcomes or sub-studies within trials (rather than trials within meta-analyses) were compared. CAM = complementary and alternative medicine; CDSR = Cochrane Database of Systematic Reviews; CVD = cardiovascular disease; IQR = interquartile range; NA = not applicable; NR = not reported

**Table S2: Methodological characteristics of included studies**

| **Study ID** | **Type of comparison** | **Assessment of study design characteristics** | **Analysis approach used** | **How non-independence of data was addressed** |
| --- | --- | --- | --- | --- |
| Abraha 2015 | Trials with versus without characteristic (within meta-analyses) | Two reviewers independently assessed all trials | Multivariable, multilevel model and meta-meta-analytic approach | Dependent trials included, but analysis adjusted to account for this |
| Als-Nielsen 2004 | Trials with versus without characteristic (within meta-analyses) | Two reviewers independently assessed all trials | Multilevel logistic regression model | No comment (dependent trials could have been included) |
| Armijo-Olivo 2015 | Trials with versus without characteristic (within meta-analyses) | Two reviewers independently assessed all trials | Meta-meta-analytic approach | Dependent trials excluded |
| Balk 2002 | Trials with versus without characteristic (within meta-analyses) | Two reviewers independently assessed all trials | Bayesian hierarchical bias model | No comment (dependent trials could have been included) |
| Bialy 2014 | Trials with versus without characteristic (within meta-analyses) | Two reviewers independently assessed all trials | Meta-meta-analytic approach | Dependent trials excluded |
| BRANDO (Savović 2012) | Trials with versus without characteristic (within meta-analyses) | Reliance on assessments by authors of included meta-epidemiological studies | Bayesian hierarchical bias model and meta-meta-analytic approach | Dependent trials excluded |
| Chaimani 2013 | Trials with versus without characteristic (within meta-analyses) | Reliance on risk of bias assessments by authors of included meta-analyses, or two reviewers independently assessed studies for which risk of bias data were not available. | Bayesian network meta-regression models | Dependent trials excluded |
| Contopoulos-Ioannidis 2005 | Trials with versus without characteristic (within meta-analyses) | Two reviewers independently assessed all trials | No modelling | No comment (dependent trials could have been included) |
| Egger 2003 | Trials with versus without characteristic (within meta-analyses) | Reliance on quality assessments by authors of included Cochrane reviews | Meta-meta-analytic approach | No comment (dependent trials could have been included) |
| Hartling 2014 | Trials with versus without characteristic (within meta-analyses) | One reviewer assessed all studies, with verification by another | Meta-meta-analytic approach | Dependent trials excluded |
| Herbison 2011 | Trials with versus without characteristic (within meta-analyses) | Two reviewers independently assessed all trials | Meta-meta-analytic approach | Dependent trials included, but analysis adjusted to account for this |
| Hrobjartsson 2012 | Outcomes with versus without characteristic (within trials) | Two reviewers independently assessed all trials | Meta-meta-analytic approach | Dependent outcomes within trials included, but analysis adjusted to account for this (in sensitivity analysis) |
| Hrobjartsson 2013 | Outcomes with versus without characteristic (within trials) | Two reviewers independently assessed all trials | Meta-meta-analytic approach | Dependent outcomes within trials included, but analysis adjusted to account for this (in sensitivity analysis) |
| Hrobjartsson 2014a | Outcomes with versus without characteristic (within trials) | Two reviewers independently assessed all trials | Meta-meta-analytic approach | Dependent outcomes within trials included, but analysis adjusted to account for this (in sensitivity analysis) |
| Hrobjartsson 2014b | Sub-studies with versus without characteristic (within trials) | Two reviewers independently assessed all trials | Meta-meta-analytic approach | Not applicable |
| Kjaergard 2001 | Trials with versus without characteristic (within meta-analyses) | Two reviewers independently assessed all trials | Logistic regression | Dependent trials excluded |
| Moher 1998 | Trials with versus without characteristic (within meta-analyses) | Two reviewers independently assessed all trials | Logistic regression | Dependent trials probably excluded |
| Nuesch 2009a | Trials with versus without characteristic (within meta-analyses) | Two reviewers independently assessed all trials | Meta-meta-analytic approach | Dependent trials excluded |
| Nuesch 2009b | Trials with versus without characteristic (within meta-analyses) | Two reviewers independently assessed all trials | Meta-meta-analytic approach | Dependent trials included, but analysis adjusted to account for this |
| Papageorgiou 2015 | Trials with versus without characteristic (within meta-analyses) | Two reviewers independently assessed all trials | Meta-meta-analytic approach | Dependent trials excluded |
| Pildal 2007 | Trials with versus without characteristic (within meta-analyses) | Two reviewers independently assessed all trials | Meta-meta-analytic approach | No comment (dependent trials could have been included) |
| Schulz 1995 | Trials with versus without characteristic (within meta-analyses) | One reviewer assessed all studies | Logistic regression | Dependent trials excluded |
| Unverzagt 2013 | Trials with versus without characteristic (within meta-analyses) | Two reviewers independently assessed all trials | Multivariable, multilevel model and meta-meta-analytic approach | Dependent trials included, no analysis to adjust for this |
| Wood 2008 | Trials with versus without characteristic (within meta-analyses) | Reliance on assessments by authors of included meta-epidemiological studies | Logistic regression and meta-meta-analytic approach | Dependent trials excluded |

**Table S3. Study design characteristics examined in included studies**

| **Study ID** | **SeqGen** | **AllocCon** | **Baseline** | **BlindPt** | **BlindPers** | **BlindOA** | **BlindDA** | **DblBlind** | **Attrition** | **SelReport** | **Other** |
| --- | --- | --- | --- | --- | --- | --- | --- | --- | --- | --- | --- |
| Abraha 2015 |  |  |  |  |  |  |  |  | X |  |  |
| Als-Nielsen 2004 | X | X |  |  |  |  |  | X | X |  |  |
| Armijo-Olivo 2015 | X | X |  |  |  |  |  |  |  |  |  |
| Balk 2002 |  | X | X | X | X | X | X | X | X |  | X |
| Bialy 2014 | X | X |  | X | X | X |  |  | X | X | X |
| BRANDO | X | X |  |  |  |  |  | X | X |  |  |
| Chaimani 2013 | X | X |  | X |  | X |  |  |  |  |  |
| Contopoulos-Ioannidis 2005 | X | X |  |  |  |  |  | X |  |  |  |
| Egger 2003 | X | X |  |  |  |  |  | X |  |  |  |
| Hartling 2014 | X | X | X | X | X | X |  |  | X | X | X |
| Herbison 2011 |  | X |  |  |  |  |  |  |  |  |  |
| Hrobjartsson 2012 |  |  |  |  |  | X |  |  |  |  |  |
| Hrobjartsson 2013 |  |  |  |  |  | X |  |  |  |  |  |
| Hrobjartsson 2014a |  |  |  |  |  | X |  |  |  |  |  |
| Hrobjartsson 2014b |  |  |  | X |  |  |  |  |  |  |  |
| Kjaergard 2001 | X | X |  |  |  |  |  | X | X |  |  |
| Moher 1998 | X | X |  |  |  |  |  | X |  |  |  |
| Nuesch 2009a |  | X |  | X |  |  |  |  |  |  |  |
| Nuesch 2009b |  |  |  |  |  |  |  |  | X |  |  |
| Papageorgiou 2015 | X |  |  |  |  |  |  |  |  |  |  |
| Pildal 2007 | X | X |  |  |  |  |  | X |  |  |  |
| Schulz 1995 | X | X |  |  |  |  |  | X | X |  |  |
| Unverzagt 2013 | X | X | X |  |  |  |  | X | X | X | X |
| Wood 2008 |  | X |  |  |  |  |  | X |  |  |  |
| **TOTAL n (%)** | **14 (58)** | **17 (71)** | **3 (13)** | **6 (25)** | **3 (13)** | **7 (29)** | **1 (4)** | **11 (46)** | **10 (42)** | **3 (13)** | **4 (17)** |

SeqGen = random sequence generation; AllocCon = allocation concealment; Baseline = baseline imbalance; BlindPt = blinding of participants; BlindPers = blinding of personnel; BlindOA = blinding of outcome assessors; DblBlind = double blinding; Attrition = incomplete outcome data, withdrawals, losses to follow-up; SelReport = selective reporting; Other = adjusting for confounders in the analyses, block randomisation in unblinded trials, switching (crossing over to other) intervention, and ineligible characteristics including early stopping and sponsorship

**Table S4. Definitions of adequate, unclear and inadequate sequence generation**

| **Study ID** | **Adequate** | **Unclear** | **Inadequate** |
| --- | --- | --- | --- |
| Als-Nielsen 2004 | Computer-generated, random number table, coin toss, drawing cards or lots, or similar stochastic method | No description | Quasi-randomized (dates, alternation or similar) |
| Armijo-Olivo 2015 | Adequate randomisation (e.g. use of a computer software, random number table and minimisation), or acceptable methods of randomisation, but less efficient than the previous category (e.g. drawing lots, envelopes, shuffling cards,  throwing a dice) | Unclear or not reported | Inappropriate methods of sequence generation (e.g. date of birth, day of admission, hospital record number) |
| Bialy 2014 | “Low risk of bias” rating for random sequence generation according to Cochrane risk of bias tool (criteria not stated) | “Unclear risk of bias” rating for random sequence generation according to Cochrane risk of bias tool (criteria not stated) | “High risk of bias” rating for random sequence generation according to Cochrane risk of bias tool (criteria not stated) |
| BRANDO (Savović 2012) | Varied across 7 included studies, but generally included computer-generated random-number, random-number table, coin toss, shuffling, other random process | No description | Varied across 7 included studies, but generally included non-randomized, quasi-randomized (dates, alternation, case records), or open random number table |
| Chaimani 2013 | Use of a random number table, a computer random number generator, coin tossing, throwing dice, restricted randomization methods such as random permuted blocks, minimization technique or similar | When there was insufficient information about the random sequence generation to permit judgement of “Low risk” or “High risk” | No description |
| Contopoulos-Ioannidis 2005 | Computer-generated, random-number table, coin or dice toss, or other method that ensures random order | No description | Alternation, case records, dates, or similar non-random method |
| Egger 2003 | Computer-generated, random-number table, or other method that ensures random order | No description | Reported as alternation or open random-number table |
| Hartling 2014 | “Low risk of bias” rating for random sequence generation according to Cochrane risk of bias tool (criteria not stated) | “Unclear risk of bias” rating for random sequence generation according to Cochrane risk of bias tool (criteria not stated) | “High risk of bias” rating for random sequence generation according to Cochrane risk of bias tool (criteria not stated) |
| Kjaergard 2001 | Computer-generated or similar | Not applicable | No description |
| Moher 1998 | Clinical trials that reported the following methods for generation of their allocation sequence: computer, random numbers table, shuffled cards or tossed coins, and minimisation | No description | Clinical trials that reported the following methods for generation of the allocation sequence: alternate assignment and assignment by odd/even birth date or hospital number |
| Papageorgiou 2015 | Adequate according to Cochrane Collaboration criteria | No description | Quasi-RCT or other inadequate random sequence generation method according to Cochrane Collaboration criteria |
| Pildal 2007 | Computer-generated sequence, random-number tables, drawing lots or envelopes, or coin toss | No description | Alternation, case record numbers, or date of birth |
| Schulz 1995 | Computer-generated random-number, random-number table, coin toss, shuffling, other random process, or minimization | No description | Non-random |
| Unverzagt 2013 | Use of random number table or generator; minimization | No description | Sequence generation based on a date or number; allocation by judgment of the clinician or preference of the participant, on the results of a laboratory test, a series of tests, or availability of the intervention |

**Table S5. Definitions of adequate, unclear and inadequate allocation concealment**

| **Study ID** | **Adequate** | **Unclear** | **Inadequate** |
| --- | --- | --- | --- |
| Als-Nielsen 2004 | Central randomization (including pharmacy-controlled), coded identical drug boxes, envelopes that were sealed, opaque and sequentially numbered, on-site locked computer system, or similar | No description | Open allocation sequence |
| Armijo-Olivo 2015 | Any type of central randomisation (e.g. a remote telephone service or a central office), or sequentially numbered, opaque and sealed envelopes | Sealed envelopes without reporting any further details, or concealment of allocation was not reported or unclear | Allocation was clearly not hidden (e.g. being based on an open list, odd or even days of the week, participant’s birth date or the team on duty at enrolment) |
| Balk 2002 | Central randomization; blinded code; coded drug containers; drugs prepared by pharmacy; or serially numbered, opaque, sealed envelopes | No description | Random-number table, cards, or method using year of birth or registration numbers |
| Bialy 2014 | “Low risk of bias” rating for allocation concealment according to Cochrane risk of bias tool (criteria not stated) | “Unclear risk of bias” rating for allocation concealment according to Cochrane risk of bias tool (criteria not stated) | “High risk of bias” rating for allocation concealment according to Cochrane risk of bias tool (criteria not stated) |
| BRANDO (Savović 2012) | Varied across 7 included studies, but generally included central randomization, numbered or coded bottles or containers, drugs prepared by pharmacy, serially numbered, opaque, sealed envelopes, or other convincing description implying concealment | No description | Varied across 7 included studies, but generally included any method where it was obvious which treatment the next patient would be allocated (alternation, case record numbers, or dates of birth) |
| Chaimani 2013 | Participants and investigators enrolling participants could not foresee assignment because one of the following, or an equivalent method, was used to conceal allocation: central allocation, sequentially numbered drug containers of identical appearance or sequentially numbered, opaque, sealed envelopes were used | When there was insufficient information about the allocation concealment to permit judgement of “Low risk” or “High risk” | No description |
| Contopoulos-Ioannidis 2005 | Central facility, central pharmacy, or with sealed and opaque envelopes | No description | Any other method that could not be classified as adequate |
| Egger 2003 | Central randomization; numbered or coded bottles or containers; drugs prepared by pharmacy; serially numbered, opaque, sealed envelopes; or other convincing description implying concealment | No description | Alternation or open random number table |
| Hartling 2014 | “Low risk of bias” rating for allocation concealment according to Cochrane risk of bias tool (criteria not stated) | “Unclear risk of bias” rating for allocation concealment according to Cochrane risk of bias tool (criteria not stated) | “High risk of bias” rating for allocation concealment according to Cochrane risk of bias tool (criteria not stated) |
| Herbison 2011 | Trials used some form of central randomization that clearly should hide the allocation, such as a remote telephone service or randomization by a pharmacy, or trials used sealed envelopes with some form of security enhancement, such as ensuring that envelopes were opaque and numbered | Trials used sealed envelopes without any further details, or that were reported as randomized without details, and also as “double blind”, or that simply said they were randomized with no further details | Trials where the allocation was clearly not hidden, for example, being based on an open list, odd or even days of the week, participant’s birth date, or the team on duty at enrolment |
| Kjaergard 2001 | Central independent unit, sealed envelopes, or similar | Not applicable | Open random number tables or similar |
| Moher 1998 | Adequate concealment was that up to the point of treatment (e.g. central randomisation) | Trials in which allocation concealment was not reported | Trials in which allocation concealment was inadequate (e.g. alternation) |
| Nuesch 2009a | The investigators responsible for patient selection and inclusion were unable to know before allocation which treatment was next, e.g. central randomization; the use of sequentially numbered, sealed, and opaque assignment envelopes; or coded drug packs | Concealment of allocation of trials, which lacked a specific statement | No description |
| Pildal 2007 | Central randomization; coded drug containers; drugs prepared by central pharmacy; serially numbered, opaque, sealed envelopes; or other convincing description implying concealment | Approach not falling into other categories | Obvious which treatment the next patient would be allocated (alternation, case record numbers, or dates of birth) |
| Schulz 1995 | Central randomization; numbered or coded bottles or containers; drugs prepared by pharmacy; serially numbered, opaque, sealed envelopes; or other convincing description implying concealment | Approach not falling into other categories | Alternation or allocation by case record number or date of birth |
| Unverzagt 2013 | Central allocation including telephonic, web-based, and pharmacy-controlled randomization; sequentially numbered drug containers of identical appearance and opaque and sealed envelopes | No description | Open random allocation schedule; assignment of envelopes without appropriate safeguards; alternation or rotation; date of birth and case record number; any other explicitly unconcealed procedure |
| Wood 2008 | Varied across 3 included studies, but generally included central randomization, numbered or coded bottles or containers, drugs prepared by pharmacy, serially numbered, opaque, sealed envelopes, or other convincing description implying concealment | No description | Varied across 3 included studies, but generally included any method where it was obvious which treatment the next patient would be allocated (alternation, case record numbers, or dates of birth) |

**Table S6. Definitions of adequate, unclear and inadequate baseline imbalance**

| **Study ID** | **Adequate** | **Unclear** | **Inadequate** |
| --- | --- | --- | --- |
| Balk 2004 | Treatment and control groups were similar in the characteristics reported | Not applicable | Treatment and control groups were not similar in the characteristics reported |
| Hartling 2014 | “Low risk of bias” rating for baseline imbalance according to Cochrane risk of bias tool (criteria not stated) | “Unclear risk of bias” rating for baseline imbalance according to Cochrane risk of bias tool (criteria not stated) | “High risk of bias rating” for baseline imbalance according to Cochrane risk of bias tool (criteria not stated) |
| Unverzagt 2013 | Groups are comparable in factors strongly related to mortality | No description | Groups non-comparable in more than one factor strongly related to mortality (differences <10%), such as age, gender, acute physiology, and chronic health evaluation scores; hemodynamics, infectious profile, underlying disease, main comorbidities, information describing multiple organ dysfunction score, and inflammation |

**Table S7. Definitions of adequate, unclear and inadequate blinding of participants**

| **Study ID** | **Adequate** | **Unclear** | **Inadequate** |
| --- | --- | --- | --- |
| Balk 2004 | Patients were reported to have been blinded. If not stated explicitly, infants and patients receiving identical-appearing treatments (active or placebo) were considered to have been blinded | Not applicable | Patients were not reported to have been blinded |
| Bialy 2014 | “Low risk of bias” rating for blinding of participants (i.e. parents) according to Cochrane risk of bias tool (criteria not stated) | “Unclear risk of bias” rating for blinding of participants (i.e. parents) according to Cochrane risk of bias tool (criteria not stated) | “High risk of bias rating” for blinding of participants (i.e. parents) according to Cochrane risk of bias tool (criteria not stated) |
| Chaimani 2013 | The authors described the study as double dummy and used identical containers or identical pills | The authors stated that the study was double-blind but there was no adequate description in the text | No description |
| Hrobjartsson 2014b | Patients were regarded as blinded when this was explicitly reported or when blinding was indicated by use of a placebo treatment (and if there was no indication of unblinding of patients) | Not applicable | Patients were regarded as nonblinded when explicitly reported to be so, or when lack of blinding was indicated by use of an untreated control group |
| Nuesch 2009a | A placebo or sham control intervention was used and experimental and control interventions were described as indistinguishable or the use of a double dummy technique was reported | No description | No description |

**Table S8. Definitions of adequate, unclear and inadequate blinding of personnel**

| **Study ID** | **Adequate** | **Unclear** | **Inadequate** |
| --- | --- | --- | --- |
| Balk 2004 | Caregivers were reported to have been blinded. Caregivers included physicians, nurses and other health practitioners in direct patient care or parents (or equivalent) of outpatient infants | Not applicable | Caregivers were not reported to have been blinded |
| Bialy 2014 | “Low risk of bias” rating for blinding of personnel according to Cochrane risk of bias tool (criteria not stated) | “Unclear risk of bias” rating for blinding of personnel according to Cochrane risk of bias tool (criteria not stated) | “High risk of bias” rating for blinding of personnel according to Cochrane risk of bias tool (criteria not stated) |

**Table S9. Definitions of adequate, unclear and inadequate blinding of outcome assessors**

| **Study ID** | **Adequate** | **Unclear** | **Inadequate** |
| --- | --- | --- | --- |
| Balk 2004 | Outcome assessors were reported to have been blinded. Outcome assessors included physicians or other health care practitioners or researchers who evaluated either patients, their records, or their laboratory or radiology tests to determine study outcomes | Not applicable | Outcome assessors were not reported to have been blinded |
| Bialy 2014 | “Low risk of bias” rating for blinding of outcome assessor according to Cochrane risk of bias tool (criteria not stated) | “Unclear risk of bias” rating for blinding of outcome assessor according to Cochrane risk of bias tool (criteria not stated) | “High risk of bias” rating for blinding of outcome assessor according to Cochrane risk of bias tool (criteria not stated) |
| Chaimani 2013 | For hard outcomes (e.g. death) blinding of outcome assessors was evaluated as “Low risk”. When outcomes were not hard, outcome assessment was judged according to the details reported. Lab outcomes were considered as “objective” and blinding of outcome assessors was judged as adequate. | The authors stated that the study was double-blind but there was no adequate description in the text | No description |
| Hartling 2014 | “Low risk of bias” rating for blinding of outcome assessor according to Cochrane risk of bias tool (criteria not stated) | “Unclear risk of bias” rating for blinding of outcome assessor according to Cochrane risk of bias tool (criteria not stated) | “High risk of bias” rating for blinding of outcome assessor according to Cochrane risk of bias tool (criteria not stated) |
| Hrobjartsson 2012 | Outcome assessors were reported to have been blinded | Not applicable | Outcome assessors were not reported to have been blinded. |
| Hrobjartsson 2013 | Outcome assessors were reported to have been blinded | Not applicable | Outcome assessors were not reported to have been blinded. |
| Hrobjartsson 2014a | Outcome assessors were reported to have been blinded | Not applicable | Outcome assessors were not reported to have been blinded. |

**Table S10. Definitions of adequate, unclear and inadequate double blinding**

| **Study ID** | **Adequate** | **Unclear** | **Inadequate** |
| --- | --- | --- | --- |
| Als-Nielsen 2004 | Described as double-blind or ≥2 key groups (patient, physician, assessor, or analyst) were blinded | No description | Single-blind or not blinded |
| Balk 2002 | Patients and either caregivers or outcome assessors were blinded | Not applicable | Any other description not classified as adequate or unclear |
| BRANDO (Savović 2012) | Varied across 7 included studies but generally described as double-blind or patients and either outcome assessor or caregiver were blinded | No description | Varied across 7 included studies but generally included single blind or not blinded |
| Contopoulos-Ioannidis 2005 | Described as double-blind or patients and either outcome assessor or caregiver were blinded | Not applicable | Not blinded, single-blind, blinding not feasible, or unclear |
| Egger 2003 | Study described as double blind | No description | Described as open or similar |
| Kjaergard 2001 | Described as double-blind and used identical placebo tablets or similar | Not applicable | Open (not blind) or described as single-blind |
| Moher 1998 | Study described as double blind | No description | Study not described as double blind, or the method of masking was described and it was inappropriate (e.g. comparison of tablet versus injection with no double dummy). |
| Pildal 2007 | Described as double-blind or patients and caregivers were reported as blinded, placebo-controlled without indication that treatments were distinguishable or investigators unblended. Patient and assessor blinding was not categorized as double-blind in this study | No description | Not blinded, single-blind, or did not fit the definition of double-blind. Patient and assessor blinding was not categorized as double-blind in this study |
| Schulz 1995 | Participants, caregivers, and outcome assessors all described as blinded | Not applicable | Descriptions not consistent with definition of double-blind, blinding not feasible, or unclear |
| Unverzagt 2013 | Blinding of participants and health-care providers, unlikely that the blinding could have been broken; nonblinding unlikely to introduce bias; method with placebo(s) or dummy technique | No description | No blinding, incomplete blinding, or absence of placebo; blinding could have been broken |
| Wood 2008 | Varied across 3 included studies but generally described as double-blind or patients and either outcome assessor or caregiver were blinded | No description | Varied across 3 included studies but generally included single blind or not blinded |

**Table S11. Definitions of adequate, unclear and inadequate attrition**

| **Study ID** | **Adequate** | **Unclear** | **Inadequate** |
| --- | --- | --- | --- |
| Abraha 2015 | Intention-to-treat (ITT) analysis reported: trials reported the phrase “intention to treat” with no apparent deviation in the description or trials that correctly described the intention to treat principle. If a trial did not use the phrase but intended to analyse the patient data according to the original allocation of the patients, then it was classified in this category. A trial that reported analysis based on both standard and deviated approaches was classified in this category. | Trials that did not refer any intention to treat approach and did not fall into the other categories | Modified intention-to-treat (mITT) analysis reported: trialists explicitly used the term “modified intention to treat” or reported a deviation from the intention to treat approach. The number and type of deviations were retrieved and deviations were classified as treatment related deviation, baseline assessment related deviation, target condition related deviation, and post-baseline assessment related deviation. |
| Bialy 2014 | “Low risk of bias” rating for incomplete outcome data according to Cochrane risk of bias tool (criteria not stated) | “Unclear risk of bias” rating for incomplete outcome data according to Cochrane risk of bias tool (criteria not stated) | “High risk of bias” rating for incomplete outcome data according to Cochrane risk of bias tool (criteria not stated) |
| BRANDO (Savović 2012) | <=20% of patients with missing outcome data | Not applicable | >20% of patients with missing outcome data |
| Hartling 2014 | “Low risk of bias” rating for incomplete outcome data according to Cochrane risk of bias tool (criteria not stated) | “Unclear risk of bias” rating for incomplete outcome data according to Cochrane risk of bias tool (criteria not stated) | “High risk of bias” rating for incomplete outcome data according to Cochrane risk of bias tool (criteria not stated) |
| Nuesch 2009b | No exclusions: there was an explicit statement that all randomized patients were included in the analysis of the outcome extracted or if the reported numbers of patients randomized and analysed on this outcome were identical | It was unclear whether exclusions from the analysis had occurred | Any exclusions: trialists explicitly reported exclusions from the analysis, or the number of patients analysed was lower than the number of patients randomized |
| Unverzagt 2013 | Reasons for missing outcome data unlikely to be related to true outcome and ineligibility was detected blinded to assignment and outcome; number and reasons of missing outcome data balanced across intervention groups; small proportion of missing outcomes (< 10%); imputation with appropriate methods | No description | Outcome-related reasons for missing outcome data; clinically relevant proportion of missing outcomes; only disease-related mortality or ‘‘as-treated’’ analysis |

**Table S12. Definitions of adequate, unclear and inadequate (selective) reporting**

| **Study ID** | **Adequate** | **Unclear** | **Inadequate** |
| --- | --- | --- | --- |
| Bialy 2014 | “Low risk of bias” rating for selective outcome reporting according to Cochrane risk of bias tool. To receive an assessment of low risk of bias, trial publications needed to report all primary and secondary outcomes in methods and results sections, and new outcomes could not be introduced within the results section of the trial. If the primary outcome, as stated in each RCT, was not included in the results section, the domain was rated as high risk of bias. | “Unclear risk of bias” rating for selective outcome reporting according to Cochrane risk of bias tool | “High risk of bias” rating for selective outcome reporting according to Cochrane risk of bias tool |
| Hartling 2014 | “Low risk of bias” rating for selective outcome reporting according to Cochrane risk of bias tool. Reviewers compared the presented results with the outcomes mentioned in the methods section of the same article. | “Unclear risk of bias” rating for selective outcome reporting according to Cochrane risk of bias tool. Reviewers compared the presented results with the outcomes mentioned in the methods section of the same article. | “High risk of bias” rating for selective outcome reporting according to Cochrane risk of bias tool. Reviewers compared the presented results with the outcomes mentioned in the methods section of the same article. |
| Unverzagt 2013 | All pre-specified outcomes reported in a pre-specified way as described in the protocol, methods, or objectives. | No description | Missing pre-specified outcomes; incomplete reporting of measurements, analysis methods, subsets, or time points of the data; missing information to patient-relevant outcomes in critically ill patients as mortality or adverse events. |

**Table S13. Definitions of adequate, unclear and inadequate study design characteristics not classified elsewhere**

| **Study ID** | **Adequate** | **Unclear** | **Inadequate** |
| --- | --- | --- | --- |
| ADJUSTING FOR CONFOUNDERS IN THE ANALYSIS | |  |  |
| Balk 2004 | If there were baseline differences between groups that could be confounders, these were examined | Not applicable | If there were baseline differences between groups that could be confounders, these were not examined |
|  | |  |  |
| BLINDING OF PARTICIPANTS/PERSONNEL | |  |  |
| Hartling 2014 | “Low risk of bias” rating for blinding of participants/personnel according to Cochrane risk of bias tool (criteria not stated) | “Unclear risk of bias” rating for blinding of participants/personnel according to Cochrane risk of bias tool (criteria not stated) | “High risk of bias” rating for blinding of participants/personnel according to Cochrane risk of bias tool (criteria not stated) |
|  | |  |  |
| BLINDING OF DATA ANALYST | |  |  |
| Balk 2004 | Studies that explicitly reported that the analysis of data was performed by individuals who were unaware of the treatment assignment | Not applicable | Data analyst was not reported to have been blinded |
|  | | |  |
| BLOCK RANDOMISATION IN UNBLINDED TRIALS | | |  |
| Hartling 2014 | “Low risk of bias” rating for block randomisation in unblinded trials according to Cochrane risk of bias tool (criteria not stated) | “Unclear risk of bias” rating for block randomisation in unblinded trials according to Cochrane risk of bias tool (criteria not stated) | “High risk of bias” rating for block randomisation in unblinded trials according to Cochrane risk of bias tool (criteria not stated) |
|  | | | |
| SWITCHING (CROSSING OVER TO OTHER) INTERVENTION | | | |
| Unverzagt 2013 | No crossover or low proportion (<10%); reasons for crossover unlikely to be related to true outcome (decision of the patient not related to health-care provider because of protocol violation); crossover balanced in numbers across intervention groups, with similar reasons across groups | No description | Reason for cross-over data likely to be related to true outcome (e.g., decision of health-care providers) with imbalance in either numbers or reasons between intervention groups (differences of proportions >10%); as-treated analysis done with substantial departure of the intervention received from that assigned at randomization |

**Table S14. Average bias and heterogeneity associated with methodological characteristics, sub-grouped by type of intervention**

| **Study design characteristic** | **Average bias (95% CI)** | **Increase in between-trial heterogeneity (95% CI)** | **Variance in average bias**  **(95% CI)** |
| --- | --- | --- | --- |
| Inadequate/unclear sequence generation (versus adequate) |  |  |  |
| Hartling 2014: All outcomes (all trials) | dSMD -0.07 (-0.22, 0.08) | NR | NR |
| Hartling 2014: All outcomes (drug trials) | dSMD 0.07 (-0.14, 0.29) | NR | NR |
| Hartling 2014: All outcomes (non-drug trials) | dSMD -0.16 (-0.38, 0.05) | NR | NR |
|  |  |  |  |
| Inadequate/unclear allocation concealment (versus adequate) |  |  |  |
| Hartling 2014: All outcomes (all trials) | dSMD 0.09 (-0.15, 0.33) | NR | NR |
| Hartling 2014: All outcomes (drug trials) | dSMD 0.03 (-0.31, 0.37) | NR | NR |
| Hartling 2014: All outcomes (non-drug trials) | dSMD 0.17 (-0.20, 0.53) | NR | NR |
| Nuesch 2009a: Subjective outcomes (all trials) | dSMD -0.15 (-0.31, 0.02) | NR | tau 0.24 |
| Nuesch 2009a: Subjective outcomes (drug trials) | dSMD -0.24 (-0.53, 0.04) | NR | tau 0.35 |
| Nuesch 2009a: Subjective outcomes (non-drug trials) | dSMD -0.05 (-0.22, 0.12) | NR | tau 0.14 |
| Nuesch 2009a: Subjective outcomes (CAM trials) | dSMD -0.52 (-0.93, -0.10) | NR | tau 0.39 |
| Nuesch 2009a: Subjective outcomes (non-CAM trials) | dSMD -0.01 (-0.10, 0.07) | NR | tau 0.00 |
|  |  |  |  |
| Inadequate/unclear baseline imbalance (versus adequate) |  |  |  |
| Hartling 2014: All outcomes (all trials) | dSMD -0.07 (-0.28, 0.14) | NR | NR |
| Hartling 2014: All outcomes (drug trials) | dSMD 0.23 (-0.37, 0.83) | NR | NR |
| Hartling 2014: All outcomes (non-drug trials) | dSMD -0.12 (-0.28, 0.03) | NR | NR |
|  |  |  |  |
| Lack of or unclear blinding of participants (versus blinded) |  |  |  |
| Hrobjartsson 2014b: Subjective outcomes (all trials) | dSMD -0.56 (-0.71, -0.41) | NA | I^2^ 60% |
| Hrobjartsson 2014b: Subjective outcomes (acupuncture trials) | dSMD -0.63 (-0.77, -0.49) | NA | I^2^ 43% |
| Hrobjartsson 2014b: Subjective outcomes (non-acupuncture trials) | dSMD -0.17 (-0.41, 0.07) | NA | I^2^ 0% |
| Nuesch 2009a: Subjective outcomes (all trials) | dSMD -0.15 (-0.39, 0.09) | NR | tau 0.26 |
| Nuesch 2009a: Subjective outcomes (drug trials) | dSMD 0.04 (-0.12, 0.19) | NR | tau 0.10 |
| Nuesch 2009a: Subjective outcomes (non-drug trials) | dSMD -0.67 (-1.04, -0.29) | NR | tau 0.10 |
| Nuesch 2009a: Subjective outcomes (CAM trials) | dSMD -0.44 (-0.94, 0.07) | NR | tau 0.50 |
| Nuesch 2009a: Subjective outcomes (non-CAM trials) | dSMD 0.04 (-0.10, 0.18) | NR | tau 0.00 |
|  |  |  |  |
| Lack of or unclear blinding of outcome assessors (versus blinded) |  |  |  |
| Hartling 2014: All outcomes (all trials) | dSMD 0.00 (-0.11, 0.11) | NR | NR |
| Hartling 2014: All outcomes (drug trials) | dSMD 0.15 (-0.10, 0.41) | NR | NR |
| Hartling 2014: All outcomes (non-drug trials) | dSMD -0.06 (-0.14, 0.03) | NR | NR |
|  |  |  |  |
| Attrition (versus no or minimal attrition) |  |  |  |
| Hartling 2014: All outcomes (all trials) | dSMD -0.09 (-0.26, 0.07) | NR | NR |
| Hartling 2014: All outcomes (drug trials) | dSMD 0.02 (-0.20, 0.23) | NR | NR |
| Hartling 2014: All outcomes (non-drug trials) | dSMD -0.17 (-0.42, 0.07) | NR | NR |
| Nuesch 2009b: Subjective outcomes (all trials) | dSMD -0.11 (-0.28, 0.05) | NR | tau 0.28 |
| Nuesch 2009b: Subjective outcomes (drug trials) | dSMD -0.16 (-0.41, 0.09) | NR | tau 0.33 |
| Nuesch 2009b: Subjective outcomes (non-drug trials) | dSMD -0.05 (-0.17, 0.07) | NR | tau 0.00 |
| Nuesch 2009b: Subjective outcomes (CAM trials) | dSMD -0.59 (-0.87, -0.31) | NR | tau 0.17 |
| Nuesch 2009b: Subjective outcomes (non-CAM trials) | dSMD -0.01 (-0.14, 0.12) | NR | tau 0.14 |
|  |  |  |  |
| High/unclear risk of bias due to selective reporting (versus low risk) |  |  |  |
| Hartling 2014: All outcomes (all trials) | dSMD -0.06 (-0.15, 0.04) | NR | NR |
| Hartling 2014: All outcomes (drug trials) | dSMD -0.13 (-0.35, 0.10) | NR | NR |
| Hartling 2014: All outcomes (non-drug trials) | dSMD -0.04 (-0.15, 0.07) | NR | NR |
|  |  |  |  |

CAM = complementary and alternative medicine, CI = confidence interval; dSMD = difference in standardised mean differences; NA = not applicable; NR = not reported; dSMD < 0 = larger effect in trials with inadequate characteristic (or at high/unclear risk of bias)
